# Semi-Empirical Astronomical Light Pollution Evaluation of Satellite Constellations

**DOI:** 10.1007/s40295-022-00358-4

**Published:** 2023-01-03

**Authors:** Doyle T. Hall

**Affiliations:** Omitron, Inc, Colorado Springs, CO USA

**Keywords:** Satellite constellations, Astronomical light pollution, Space vehicles, Telescopes

## Abstract

Several commercial organizations have recently launched or plan to launch constellations containing thousands of satellites. Such large constellations potentially adversely affect astronomical observations. This study formulates a set of indicators that assess the impact of light pollution from different constellations on ground-based visible band astronomy. These include the statistically expected number of visible and sunlit satellites above ground-based observers, as well as the number that are also expected to be brighter than the currently recommended limit for constellation satellites. The latter indicator provides a consolidated means to evaluate the potential for a constellation to affect ground-based astronomy too severely, by simultaneously accounting for the effects of constellation population, orbital distribution as well as brightness magnitude and variability. For existing constellations, the evaluation process incorporates actual satellite photometric brightness measurements, which are becoming increasingly available in web-accessible databases and repositories. For proposed constellations, a semi-empirical method allows rough approximations of pre-launch light pollution levels, based on observed brightness distributions observed of currently orbiting analog satellites.

## Summary

Several commercial organizations have recently launched or plan to launch constellations containing thousands of satellites. Such large constellations potentially adversely affect astronomical observations. This study formulates a set of indicators that assess the impact of light pollution from different constellations on ground-based visible band astronomy. These include the statistically expected number of visible and sunlit satellites above ground-based observers, as well as the number that are also expected to be brighter than the currently recommended limit for constellation satellites. The latter indicator provides a consolidated means to evaluate the potential for a constellation to affect ground-based astronomy too severely, by simultaneously accounting for the effects of constellation population, orbital distribution as well as brightness magnitude and variability. For existing constellations, the evaluation process incorporates actual satellite photometric brightness measurements, which are becoming increasingly available in web-accessible databases and repositories. For proposed constellations, a semi-empirical method allows rough approximations of pre-launch light pollution levels, based on observed brightness distributions observed of currently orbiting analog satellites.

## Introduction

Several commercial organizations have recently launched or plan to launch constellations containing hundreds or even thousands of Earth-orbiting satellites, which can adversely affect astronomical observations [1–11]. Adverse effects arise from sunlight reflected from the satellites (e.g., in the visible and near-IR spectral bands), emitted radiation (e.g., in the thermal-IR and radio bands), as well as from occultations, in which constellation satellites block the light from astronomical objects. However, it is important to note that the deployment of such large constellations creates a wide variety of new risks, in addition to those to ground-based astronomy. Boley and Byers [12] discuss how the recent rapid development of large constellations risks multiple tragedies of the commons including: increased satellite collisions and runaway on-orbit fragmentation, atmospheric pollution from both rocket launches and materials deposited by re-entering satellites, and human casualties and other damage caused by surface impacts after space object re-entry.

The primary missions of the NASA Conjunction Assessment Risk Analysis (CARA) team include assessing and mitigating collision risks for a specific set of high value Earth-orbiting satellites [13], as well as establishing and documenting associated methods [14]. However, the “NASA Spacecraft Conjunction Assessment and Collision Avoidance Best Practices Handbook” also provides guidance on other related matters, including the following specific recommendation for owners and operators of satellite constellations [14]:

“If the constellation, given its population, orbit, and constituent satellites, is likely to affect ground-based astronomy, reassign the satellite orbits or modify the satellite construction to eliminate this effect.”

This study aims to develop a single composite indicator, or small set of indicators, designed specifically to help make this light pollution assessment. The analysis specifically focuses on measuring constellation light pollution risks for ground-based astronomical observations conducted in the visible and near-IR spectral bands. The methodology is semi-empirical, incorporating brightness magnitudes of constellation satellites measured by ground-based sensors, combined with a semi-analytical model of the associated effects on astronomical observations.

Evaluations to assess light pollution risk must often be made in the definition phase of a constellation’s development, in the absence of a specific or durable spacecraft design, or while the number and orbital distribution of satellites is being established in design trade studies. In such pre-launch situations, using high fidelity modeling and simulation methods to estimate the impact of the deployed constellation is sometimes not feasible, because the required parameters are not available. To address this issue, this study also discusses the benefits and limitations of using a semi-empirical approach for proposed constellations based on photometric observations of currently orbiting analog satellites.

### Overview of Astronomical Light Pollution from Large Constellations

The astronomical community has analyzed the effects of satellite constellations in detail, and developed a wide array of quantitative methods to measure their impact (see [1–12] and references therein). Extensive discussions on astronomical light pollution have occurred within the last few years at several international forums — including the two *SatCon* conferences [3, 8] and the two *Dark and Quiet Skies for Science and Society* conferences [7, 10]. Recently, Bassa et al. [11] formulated a set of semi-analytical methods to evaluate the impact of constellations for a variety of optical and near-IR observation modalities. These efforts have produced a wide array of indicator functions to evaluate light pollution, including: the number of visible or illuminated constellation satellites above an observatory, their apparent and range-normalized stellar magnitudes, their contribution to overall sky brightness levels, their capacity and frequency of glinting in reflected sunlight, and their angular drift rates across sensor apertures and detector arrays. These quantities can change significantly as a function of time and ground-based location, which increases the number of potential ways to measure astronomical light pollution levels even further.

### Recommended Brightness Threshold for Constellation Satellites

The brightness of individual constellation satellites represents a key consideration when evaluating light pollution. In order to preserve the visual appearance of the night sky and limit adverse effects on ground-based observations, the consensus of the astronomical community is to keep any satellite fainter than about 7th visual magnitude [3, 7–10]. This analysis adopts the specific brightness limit recommended in the *SatCon-1 Workshop Report* [3], that low-Earth orbit (LEO) constellation satellites should be no brighter than a threshold visual magnitude given by

1 $${\mathbb{M}}(h)=7.0+2.5{\mathrm{log}}_{10}\left(\frac{h}{550\,\text{km}}\right)$$

with $h$ indicating the orbital altitude. Section 4 of this study presents ground-based brightness measurements for a selection of currently orbiting constellation satellites, and compares both the median magnitude and the level of variability to this recommended limit. (Note: this discussion often refers to magnitudes brighter than the threshold given by Eq. (1) as “brighter-than-recommended” for brevity.) Sect. 5 then formulates semi-analytical expressions that use measured brightness distributions to estimate the time-averaged number of brighter-than-recommended satellites above ground-based observers—a composite light pollution indicator that incorporates the effects of constellation population, orbital distribution as well as brightness magnitude and variability [15].

### Key Light Pollution Considerations and Parameters

Many parameters contribute to a constellation’s overall potential to impact ground-based optical astronomy [1–12]. These comprise two broad classes: constellation parameters and observational parameters. Constellation parameters include the total number of satellites, their distribution of altitudes, inclinations and other orbital parameters, as well as the magnitude and variability of the brightness of the individual satellites. Observation parameters include the observatory location, the time of the measurements, the spectral band, the atmospheric extinction, the sensor modality (e.g., wide-field imaging, narrow-field imaging, spectroscopy, etc.), the required exposure times, as well as the capability of the sensors to schedule and perform mitigations such as mid-exposure interruptions.

Reflected sunlight dominates the brightness of typical satellites in the visible and near-IR spectral bands. For this reason, another key parameter must be considered when evaluating the potential impact of constellations: the solar depression angle, $\alpha$, which is also denoted as SDA in the analysis. This angle measures how far the Sun is below a ground-based observer’s local horizon, (i.e., the negative of the solar altitude or elevation angle). Sunset and sunrise occur when $\alpha$ = 0, and three periods of twilight are defined as follows:

- Civil twilight: 0° ≤ $$\alpha$$  < 6°
- Nautical twilight: 6° ≤ $$\alpha$$  < 12°
- Astronomical twilight:12° ≤ $$\alpha$$  < 18°

Astronomical night spans periods when $\alpha$ ≥ 18°, which is often the most valuable and productive time to conduct ground-based observations, especially for faint targets, because of the low level of sky foreground contamination. As the SDA increases into astronomical night, a steadily decreasing number of LEO satellites remain sunlit as they enter Earth’s shadow above a ground-based observer [11, 15]. For this reason, this analysis conservatively evaluates light pollution based on the statistically expected number of brighter-than-recommended satellites above a ground-based observer estimated for $\alpha$ = 18°, which corresponds to the maximum value that occurs during astronomical nighttime periods.

Section 5 shows that, in addition to the SDA, the number of brighter-than-recommended constellation satellites above ground-based observers depends on the latitude of the observatory, ${\beta }_{o}$, and the subsolar latitude, ${\beta }_{s},$ which varies between ± 23.5° during each year. Again, the analysis conservatively evaluates constellation light pollution levels based the maximum expected number of brighter-than-recommended satellites estimated as a function of these angles.

### Objectives and Methodology

The main objective of this study is to develop a method to evaluate constellation light pollution levels for ground-based visible and near-IR spectral band observations conducted during astronomical nighttime periods. The methodology combines empirical constellation brightness distributions with a semi-analytical model of the associated effects on ground-based observers, applied to both existing and proposed constellations.

## Constellation Parameters

Current and proposed constellations typically contain a large number of identical (or nearly identical) LEO satellites. Generally, constellations comprise multiple orbital “shells,” each with a distinct altitude and inclination, which are further subdivided into multiple orbital planes [11]. Table 1 shows parameters for five representative large constellations studied as example cases in this analysis. These include the 1st and 2nd Generations of the SpaceX Starlink constellations, and Phase 1 and 2 of the OneWeb constellations. (This analysis uses parameters for these four constellations as reported by Bassa et al. [11].) The current partially deployed status of the Starlink 1st Gen. and OneWeb Phase 1 constellations allow the evaluation process to incorporate actual ground-based observations of operational on-orbit satellites. For comparison purposes, the analysis also uses actual ground-based observations of the less populous Iridium 2nd Generation constellation, which currently has 66 operational satellites and 9 on-orbit spares, orbiting in a single shell at an altitude of 780 km [16]. Table 1 also lists the threshold magnitudes calculated using Eq. (1) for each component shell of theses constellations. Finally, Table 1 lists two hypothetical single satellites, used to illustrate how evaluated light pollution levels for bright isolated satellites compare to multi-satellite constellations. Specifically, the “Low Incl. Bright Sat.” represents an object occupying a low inclination orbit similar to that of the Hubble Space Telescope; the “High Incl. Bright Sat.” represents an object in an orbit similar to that of a typical sun synchronous satellite [17]. For simplicity, the analysis assumes that both of these hypothetical satellites are brighter than the threshold given in Eq. (1) under all circumstances. Also for illustrative purposes, the analysis sometimes discusses the first shell of the Starlink 1st Gen. constellation as if it were an isolated constellation.

**Table 1:** Parameters for the studied constellations

Constellation name (and component shell *j*)	Number of satellites ${N}_{c}$ or ${N}_{c,j}$	Orbital altitude (km) ${h}_{j}$	Inclination (°) ${i}_{j}$	Threshold magnitude ${\mathbb{M}}({h}_{j})$
Starlink 1st Generation	11,926 total	335.9 – 570	42.0 – 97.6	6.46 – 7.04
shell 1	1584	550	53.0	7.00
shell 2	1584	540	53.2	6.98
shell 3	720	570	70.0	7.04
shell 4	348	560	97.6	7.02
shell 5	172	560	97.6	7.02
shell 6	2493	335.9	42.0	6.46
shell 7	2478	340.8	48.0	6.48
shell 8	2547	345.6	53.0	6.50
Starlink 2nd Generation	30,000 total	328 – 614	30.0 – 148.0	6.44 – 7.12
shell 1	7178	328	30.0	6.44
shell 2	7178	334	40.0	6.46
shell 3	7178	345	53.0	6.49
shell 4	2000	360	96.9	6.54
shell 5	1998	373	75.0	6.58
shell 6	4000	499	53.0	6.89
shell 7	144	604	148.0	7.10
shell 8	324	614	115.7	7.12
OneWeb Phase 1	1980	1200	87.9	7.85
OneWeb Phase 2	6372	1200	55.0 – 87.9	7.85
shell 1	1764	1200	87.9	7.85
shell 2	2304	1200	40.0	7.85
shell 3	2304	1200	55.0	7.85
Iridium 2nd Generation	75	780	86.4	7.38
Low Incl. Bright Sat	1	540	28.5	6.98
High Inc. Bright Sat	1	700	88	7.26

## Observed Constellation Satellite Brightnesses

The Starlink 1st Gen., OneWeb Phase 1, and Iridium 2nd Gen. constellations have been partially or completely deployed. Extensive photometric observations of many of these constellation satellites have been conducted [4, 11], including those acquired by the Mini-MegaTORTORA (MMT) automated observatory in Russia, which measures clear-filter photometric magnitudes, which are roughly equivalent to measurements conducted in the Johnson-Cousins V band-pass [18, 19]. Observational analyses using MMT data have been conducted for both the OneWeb constellation [20], and for the Starlink constellation [19, 21], with the latter including the “VisorSat” design for Starlink satellites, incorporating specific manufacturing modifications to reduce the amount of sunlight reflected towards ground-based observers [10, 22]. (MMT temporal light-curve data for these constellations, and an extensive array of other satellites are available from the http://mmt9.ru/satellites/ website [18–21].)

**Fig. 1:**
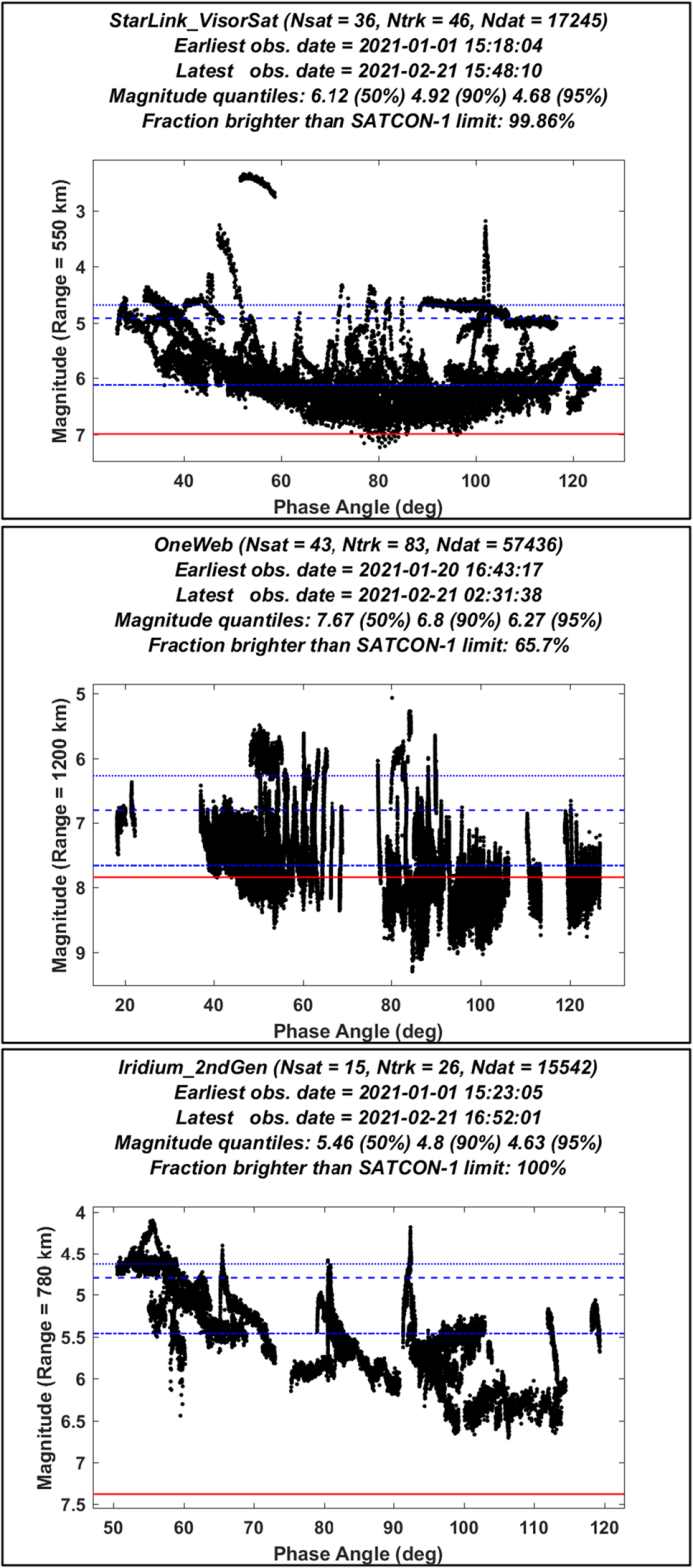
Zenith range-normalized magnitudes measured by the MMT facility [18–20] for satellites from the first shell of the Starlink 1st Gen. constellation (top), the OneWeb Phase 1 constellation (middle), and the Iridium 2nd Gen. constellation (bottom), plotted as a function of phase angle. Horizontal red lines show the corresponding recommended maximum brightness magnitudes from Eq. (1). The horizontal blue lines show the 50%, 90% and 95% quantiles of the observed distributions

Figure 1 shows multiple MMT light-curves for currently deployed Starlink 1st Gen., OneWeb Phase 1, and Iridium 2nd Gen. constellation satellites, plotted as a function of solar phase angle (i.e., the observer-satellite-sun angle). Each data set includes observations of 15 or more distinct constellation satellites, and a much larger number of individual photometric measurements (each represented by a black dot). Each individual light-curve (i.e., track) represents a single traversal of a satellite over the observatory, and appears in Fig. 1 as a coherent, nearly continuous group of black dots (one such light-curve can be identified relatively easily in the upper part of the top panel). The data sets plotted in Fig. 1 are not intended to represent a comprehensive profile of ground-based photometric brightnesses of the constellation satellites. Instead, they represent a subset the available MMT data, observed during the first two months of 2021, used here to establish light pollution evaluation methods. Light-curves for Starlink were restricted to VisorSat design satellites [22] deployed into the first shell of Starlink 1st Gen. constellation at an altitude of 550 km. In addition, data sets for all three constellations only include light-curves measured after the satellites had finished maneuvering up to their final, operational orbital altitudes. The analysis imposes no other data selection criteria, in an attempt to obtain a representative initial statistical characterization of the overall brightness and variability of satellites in each constellation’s operational configuration.

The magnitudes plotted in Fig. 1 obviously correspond to times when the constellation satellites were bright enough for the MMT sensor to detect. However, there likely were other times that the satellites were too dim for detection. This means the brightness distributions shown in Fig. 1 likely are somewhat biased, due the exclusion of these non-detection events. For sensors that record the occurrences of non-detections, and provide the means to estimate associated upper-limit brightness values, statistical methods such as the Kaplan–Meier estimator provide a method to account for this biasing effect [23]. Unfortunately, such non-detection information is not readily available for the MMT data shown in Fig. 1, so this analysis neglects this biasing effect. Ideally, however, such non-detections and associated upper-limits should be accounted for in future analyses, if possible—an important consideration for on-going constellation photometry compilation and sharing efforts, such as the “SatHub” data repository [8]. Preliminary analysis indicates that it would be straightforward to extend the semi-empirical method presented here to incorporate the Kaplan–Meier estimator method [23].

### Normalized Satellite Magnitudes

Each plot in Fig. 1 shows photometric brightnesses in stellar magnitudes, normalized to a constant satellite range as well as to a solar range of one astronomical unit (AU = 1.496 × 10^8^ km). Specifically, the magnitude of a photometric observation normalized to an observer-to-satellite range of ${\rho }_{n}$ can be calculated from the calibrated exo-atmospheric MMT magnitude, $m$, using the following adjustment [11]

2 $${M}_{n}=m-5{\mathrm{log}}_{10}\left(\rho /{\rho }_{n}\right)-5{\mathrm{log}}_{10}\left({\rho }_{s}/\mathrm{AU}\right)$$

with $\rho$ indicating the observer-to-satellite range at the time of the observation, and ${\rho }_{s}$ the corresponding Sun-to-satellite range. The adjustment for solar range is typically much smaller than that for observer range. Note, each plot in Fig. 1 uses the altitude of the observed constellation satellites as the normalization range, i.e., ${\rho }_{n}=h$. For this reason, this analysis sometimes refers to ${M}_{n}$ as the “zenith range-normalized magnitude” or just the “zenith magnitude” for brevity, which has also been used in previous analyses to describe the brightness distributions of constellation satellites [1, 11, 20, 21].

The solid red horizontal lines in Fig. 1 show the maximum recommended brightness threshold magnitudes for the three observed constellations, calculated using Eq. (1). Notably, for all three constellations, most or all measured zenith magnitudes are brighter than the recommended threshold. As will be discussed later, the primary light pollution indicator used in this analysis corresponds to the time-averaged number of brighter-than-recommended satellites above ground-based observers, estimated as a statistical expectation value based on the observed brightness distributions. Notably, this semi-empirical indicator would equal zero if there were no photometric measurements brighter than the recommended threshold, which would be the case if all of the black dots were to appear below the red lines in the panels in Fig. 1. However, because of the significant number of measurements that exceed the recommended brightness threshold for each of these constellations, the analysis indicates non-zero levels of light-pollution in each case.

### Satellite Brightness Variations

The brightness of a manufactured satellite in reflected sunlight represents a complex function of a large number of parameters, broadly divided into three categories: observational, environmental and satellite parameters. Observational parameters include time-dependent geometrical variations, such as observer-to-satellite range, solar phase angle, etc. Usually, environmental parameters, such as the illuminating solar flux or extinction due to atmospheric absorption, are either well known, or can be accounted for reasonably accurately as part of the photometric calibration process. Satellite parameters further subdivide into two categories: attitude and body parameters [24]. Attitude parameters specify the time-dependent inertial orientation of the satellite itself and its articulating components, and provide the means to convert between the inertial reference frame and the body-fixed reference frame. Body parameters comprise all of the information required to calculate the radiant intensity of the object from within the body-fixed reference frame, including parameters describing the shape of the satellite, as well as the reflectance characteristics of the materials covering the outer surfaces. As Fig. 1 indicates, this large number of satellite-specific parameters along with variations in observation and illumination geometry together create significant variability in ground-based range-normalized brightness measurements for actual satellites. Modeling or analyzing the nature and causes of these variations can be labor intensive, and is beyond the scope of this study. Instead, this analysis uses an approach that relies on statistically characterizing the observed brightness distributions of constellation satellites.

### Statistical Characterization of Constellation Satellite Magnitudes

The representative data sets plotted in Fig. 1 empirically demonstrate that photometric brightnesses vary significantly for all three of the observed constellations, and are likely to vary similarly for future constellations. Specifically, zenith magnitudes vary with time in two ways: both within individual light-curves—often caused by occasional specular glints of sunlight from satellite components—as well as over longer time scales, i.e., from light-curve to light-curve—which can be caused by satellite attitude or shape configuration changes. For instance, Starlink satellites employ at least two different attitude/shape configurations, called the “open-book” and “shark-fin” modes, known to create significantly different brightnesses recorded by ground-based sensors [3, 22].

Figure 1 indicates the existence of brightness variation trends as a function of solar phase-angle, which are measurable but do not contribute significantly to the overall variability, except for perhaps in the Iridium 2nd Gen. constellation. This analysis makes no effort to account or correct for variations with phase angle, although this (and other observational geometric considerations) could be incorporated into future studies using a similar semi-empirical methodology.

The analysis statistically characterizes constellation brightnesses using three quantiles of the observed distributions: the 50% quantile (i.e., the median magnitude), for which 50% of the measurements have dimmer magnitudes, as well as the 90% and 95% quantiles, which serve as dual indicators of both the prevalence and amplitude of upward brightness variations. Figure 1 shows these three quantile magnitudes (denoted here as ${M}_{z}^{50}$, ${M}_{z}^{90}$ and ${M}_{z}^{95}$, respectively) plotted as horizontal blue lines over the photometric data. In general, for constellations that have few upward excursions in brightness, whether due to less frequent glinting or the use of less reflective attitude/shape configurations, the 90% and 95% quantiles should not differ too greatly from the median value. However, for constellation satellites that glint sunlight more often, or that frequently employ brighter attitude/shape configurations, these quantiles will differ from the median value more significantly.

#### Starlink VisorSat Satellite Brightnesses

The median zenith range-normalized magnitude of the first shell of the Starlink 1st Gen. constellation estimated from the data plotted in Fig. 1 is ${M}_{n}^{50}$ ≈ 6.12, which is about 0.2 magnitudes fainter than the average value of 5.91 reported in earlier, more extensive studies of MMT VisorSat observations [19, 21]. Notably this represents a relatively small difference compared to the overall level of variation for the combined data. The Starlink 90% quantile is ${M}_{n}^{90}$ ≈ 4.92, which is 1.2 magnitudes brighter than the median. This difference is due, in part, to the two or three brightest Starlink VisorSat light-curves plotted in Fig. 1, which are each significantly brighter than ${M}_{n}^{50}$ throughout their entire durations. Figure 1 also indicates that more than 99% of the zenith magnitudes for the first Starlink shell are brighter than the recommended threshold of ${\mathbb{M}}$ = 7. (Note, this analysis denotes the brighter-than-recommended fraction of zenith magnitudes as ${f}_{0}$)

#### OneWeb Satellite Brightnesses

The median for the OneWeb constellation satellites estimated from the data plotted in Fig. 1 is ${M}_{n}^{50}$ ≈ 7.67, about 0.1 magnitude brighter than the average value of 7.58 reported in an earlier MMT study [20], again a relatively small difference compared to the overall level of variation. The OneWeb 90% quantile is ${M}_{n}^{90}$ ≈ 6.80, about 0.9 magnitudes brighter than the median. Notably, many of the individual light-curves for OneWeb satellites plotted in Fig. 1 vary by 1.5 magnitudes or more during their duration. Figure 1 indicates that ${f}_{0}$ = 65.7% of the measured zenith magnitudes for OneWeb are brighter than the recommended threshold of ${\mathbb{M}}$ = 7.85.

#### Iridium 2nd Generation Satellite Brightnesses

The median for the Iridium 2nd Gen. constellation estimated from the data in Fig. 1 is ${M}_{n}^{50}$ ≈ 5.46, and the 90% quantile is ${M}_{n}^{90}$ ≈ 4.80, about 0.7 magnitudes brighter. This makes individual Iridium satellites the brightest among the three studied constellations, as represented by the ${M}_{n}^{50}$ and ${M}_{n}^{90}$ indicators. Figure 1 also indicates that ${f}_{0}$ = 100% of the zenith magnitudes for this constellation are brighter than the recommended threshold of ${\mathbb{M}}$ = 7.38.

## Formulation of Indicators for Light Pollution Evaluation

This section formulates semi-analytical expressions for a set of three composite indicators that assess in different ways the potential light pollution risk of a constellation. The first two represent the time-averaged number of visible and sunlit satellites expected to be somewhere in the sky above ground-based observers, as discussed and formulated in previous analyses (e.g., [1] and [11]). The methodology presented here formulates these two quantities based on the spatial probability density function (PDF) of orbiting satellites derived analytically in 1981 by Kessler [25], and results in semi-analytical expressions consistent with those derived more recently by Bassa et al.[11]. The analysis then extends the derivation to estimate the statistically expected number of brighter-than-recommended satellites above ground-based observers, the primary indicator used in this analysis to evaluate constellation light pollution.

### The Number of Visible and Illuminated Constellation Satellites Above a Ground-based Observer

In this analysis, a “visible” object represents any constellation satellite that is somewhere in the sky above a ground-based observer (i.e., above the observer’s local horizon). When the sun is below the horizon (i.e., at times when the SDA is in the range 0 < $\alpha$  ≤ 90°), only a fraction of visible LEO satellites are also illuminated by the sun. This section formulates expressions for the statistically expected, time-averaged number of visible and sunlit constellation satellites above a ground-based observer.

#### Line-of-Sight Integration through the Time-Averaged Spatial Density of Satellites

The statistically expected number of visible satellites above a ground-based observer, ${N}_{v}$, can be estimated by first integrating the long-term average spatial density of the constellation satellites along the line of sight (LOS) defined by zenith angle,$\theta$, and azimuth angle, $\phi$, and then integrating over solid angle, as follows

3 $${N}_{v}={\int }_{0}^{\pi /2}d\theta {s}_{\theta }{\int }_{0}^{2\pi }d\phi {\int }_{A}^{B}d\rho {\rho }^{2} S({\varvec{r}})$$

with $\rho$ indicating the range from the observer along the LOS. (Note: this equation denotes the sine of the zenith angle as ${s}_{\theta }=\mathrm{sin}(\theta )$, a compact notation used for both sine and cosine functions throughout this analysis.) The innermost (i.e., rightmost) integral represents integration along the LOS, with lower and upper range limits, $A$ and $B$, corresponding to the bounds of the volume containing the constellation satellites (as described in more detail later). The two outer integrals over $\theta$ and $\phi$ in Eq. (3) represent integration over solid angle, i.e., over the entire sky as seen by the observer. Taken together, the three integrals in Eq. (3) also represent integration over the volume above the observer’s horizon. The integrand function, $S({\varvec{r}})$, represents the time-averaged spatial density of constellation satellites (also described in more detail later), expressed as a function of the inertial position vector along the LOS, ${\varvec{r}}$, as given by the following parametric equations

4a $${\varvec{r}}={\varvec{r}}\left(\rho ,\phi , \theta \right)={{\varvec{r}}}_{o}+\rho \widehat{l}\left(\phi , \theta \right)$$

and

4b $$\widehat{l}=\widehat{l}\left(\phi , \theta \right)={\widehat{{\varvec{n}}}}_{o}{c}_{\phi }{s}_{\theta }+{\widehat{{\varvec{e}}}}_{o}{s}_{\phi }{s}_{\theta }+{\widehat{{\varvec{r}}}}_{o}{c}_{\theta }$$

In these equations, ${{\varvec{r}}}_{o}$ indicates the position of the ground-based observer in the Earth-centered inertial (ECI) frame of reference, and $\widehat{l}$ indicates the unit vector along the LOS, which is expressed using the observer’s local horizon coordinate system north (${\widehat{{\varvec{n}}}}_{o}$), east (${\widehat{{\varvec{e}}}}_{o})$ and radial (${\widehat{{\varvec{r}}}}_{o})$ unit vectors.

For a given constellation, the number of visible satellites above an observer depends on three observational parameters: the time of the observation, $t$, and the geocentric longitude and latitude of the observatory, $({\lambda }_{o},{\beta }_{o})$. This dependence can be expressed functionally as ${N}_{v}={N}_{v}(t,{\lambda }_{o},{\beta }_{o})$, but these variables are usually suppressed in this analysis for brevity.

#### The Number of Sunlit Constellation Satellites Above a Ground-based Observer

The expression for the expected number of satellites also illuminated by the Sun is similar to Eq. (3)

5 $${N}_{i}={\int }_{0}^{\pi /2}d\theta {s}_{\theta }{\int }_{0}^{2\pi }d\phi {\int }_{A}^{B}d\rho {\rho }^{2} S\left({\varvec{r}}\right) {I}_{s}({\varvec{r}},{{\varvec{r}}}_{s})$$

This integrand contains an extra factor, the binary function ${I}_{s}\left({\varvec{r}},{{\varvec{r}}}_{s}\right)$, which indicates the solar illumination status of a satellite at the ECI position ${\varvec{r}}$ along the LOS, such that ${I}_{s}$ = 1 when sunlit, and ${I}_{s}$ = 0 otherwise. This function also depends on the ECI position of the sun, ${{\varvec{r}}}_{s}$. Approximating the Earth as a sphere and the Sun as a point-source illuminator allows straightforward analytical evaluation of ${I}_{s}\left({\varvec{r}},{{\varvec{r}}}_{s}\right)$. Analytical evaluation is also possible but less straightforward for an oblate Earth (with polar radius ~ 22 km less than that of the equator) and for a solar disk of finite angular extent (which creates a penumbral shadow), but analysis indicates that these relatively minor effects do not affect the results significantly.

Like ${N}_{v}$, the number of sunlit satellites above an observer depends on the time of the observation, and the longitude and latitude of the observatory, i.e., ${N}_{i}={N}_{i}(t,{\lambda }_{o},{\beta }_{o})$. At sunrise or sunset (i.e., at times when SDA = 0) all satellites above an observer are sunlit, so ${N}_{i}={N}_{v}$. At the edge of astronomical night (when SDA = 18°), ${N}_{i}\le {N}_{v}$ for LEO constellations. Beyond that, ${N}_{i}$ tends to decrease quickly with increasing SDA for lower altitude constellations; however, ${N}_{i}$ can remain almost as large as ${N}_{v}$ well into astronomical night for higher altitude constellations [11, 15].

#### The Kessler Time-Averaged Spatial Density of Satellites

In 1981, for the purposes of collision probability estimation, Kessler [25] derived an expression for the time-averaged volumetric PDF of a single satellite orbiting an oblate central body

6 $$K(r,\beta ;a,e,i) = \left\{ {\begin{array}{*{20}c} {(2\pi^{3} ra[(r - q)(Q - r)(s_{i}^{2} - s_{\beta }^{2} )]^{{{1 \mathord{\left/ {\vphantom {1 2}} \right. \kern-\nulldelimiterspace} 2}}} )^{ - 1} } & {if\;q \le r \le Q\;\&\; s_{\beta }^{2} \le s_{i}^{2} } \\ 0 & {{\text{otherwise}}} \\ \end{array} } \right.$$

with $r=|{\varvec{r}}|$ denoting the inertial-frame radial distance and $\beta =\mathrm{arcsin}(\widehat{{\varvec{z}}}\bullet {\varvec{r}}/ r)$ the geocentric latitude. The PDF also depends on $(a,e,i)$, the orbit’s semi-major axis, eccentricity and inclination, respectively, and is expressed conveniently in terms of the perigee distance $q=a(1-e)$ and apogee distance $Q=a(1+e)$. The orbital semi-major axis is $a={R}_{e}+h$, with ${R}_{e}$ equal to the equatorial radius of the Earth, and $h$ the satellite’s altitude. The Kessler approximation idealizes the orbit as being perturbed only by the central body’s J_2_ gravitational term, and represents an average over time scales much longer than the J_2_-induced cyclical period of the right ascension of the ascending node [17, 25]. Notably, even though the function given in Eq. (6) diverges on the boundaries of the Kessler density volume, defined by $q\le r\le Q$ and ${s}_{\beta }^{2}\le {s}_{i}^{2}$, integrating the PDF over the entire volume yields a finite result of one (which can be done analytically).

The Kessler PDF provides an estimate of the time-averaged spatial density of a single shell of a constellation

7 $${S}_{j}\left({\varvec{r}}\right)={N}_{c,j} K\left(r,\beta ;{a}_{j},{e}_{j},{i}_{j}\right)$$

with ${N}_{c,j}$ denoting the number of satellites in shell number *j*, so that ${N}_{c}={\sum }_{j}{N}_{c,j}$ is the total number in the constellation, and $S\left({\varvec{r}}\right)={\sum }_{j}{S}_{j}\left({\varvec{r}}\right)$ is the long-term average spatial density of constellation satellites at location ${\varvec{r}}$. Combining eqs. (5) and (7) yields the number of illuminated satellites in the *j*th shell above a ground-based observer

8 $${N}_{i,j}={\int }_{0}^{\pi /2}d\theta {s}_{\theta }{\int }_{0}^{2\pi }d\phi {\int }_{{A}_{j}}^{{B}_{j}}d\rho {\rho }^{2} {S}_{j}\left({\varvec{r}}\right) {I}_{s}({\varvec{r}},{{\varvec{r}}}_{s})$$

with the total given by a sum over all constellation shells, ${N}_{i}={\sum }_{j}{N}_{i,j}$. The range integration limits, ${A}_{j}$ and ${B}_{j}$, represent intersections of the LOS with the inner and outer spheres that bound the *j*th shell, which have radii equal to the perigee and apogee distances, i.e., ${q}_{j}$ and ${Q}_{j}$. Approximating Earth’s figure as a sphere with radius ${R}_{e}$ provides the lower range limit

9 $${A}_{j}=\varrho \left({q}_{j},\theta \right)={\left[{q}_{j}^{2}-{({R}_{e}{s}_{\theta })}^{2}\right]}^{1/2}- {R}_{e}{c}_{\theta }$$

(Note: this formulation introduces the function $\varrho$ here to use in later expressions.) Similarly, the upper integration limit for the shell is ${B}_{j}=\varrho \left({Q}_{j},\theta \right)$. The overall range limits for Eqs. (3) and (5) are $A=\mathrm{min}({A}_{j})$ and $B=\mathrm{max}({B}_{j})$

#### Approximating the Line-of-Sight Integral

The innermost integral over the LOS range $\rho$ in Eq. (8) can be evaluated analytically by changing the integration variable to $u=(r-{a}_{j})/({a}_{j} {e}_{j})$, and then approximating the resulting integrand to first order in ${e}_{j}$. These transformations yield the following expression for the number of sunlit satellites in the *j*th shell above the observer

10 $${N}_{i,j}\approx \frac{{N}_{c,j}}{2{\pi }^{2}{a}_{j}}{\int }_{0}^{\pi /2}d\theta \left\{\frac{{s}_{\theta } \; {\varrho }^{2}\left({a}_{j},\theta \right)}{ {\left[{a}_{j}^{2}-{({R}_{e}{s}_{\theta })}^{2}\right]}^{1/2}}{\int }_{0}^{2\pi }d\phi \left[\frac{U\left({w}_{j}\right) {I}_{s}({{\varvec{r}}}_{j},{{\varvec{r}}}_{s})}{\sqrt{{w}_{j}}}\right]\right\}$$

with ${{\varvec{r}}}_{j}=\boldsymbol{ }{\varvec{r}}\left(\varrho ({a}_{j},\theta ),\phi , \theta \right)$ given by Eq. (4), and ${w}_{j}={s}_{i}^{2}-{(\widehat{{\varvec{z}}}\bullet {{\varvec{r}}}_{j}/ {r}_{j})}^{2}$, both introduced here for brevity. The unit step function in the integrand, $U\left(x\right)$, accounts for the fact that the Kessler density equals zero for latitudes higher than the orbital shell’s inclination. Setting ${I}_{s}\left({{\varvec{r}}}_{j},{{\varvec{r}}}_{s}\right)$ = 1 in Eq. (10) yields the expression for ${N}_{v,j}$, the number of visible satellites in the *j*th shell. A careful inspection of Eq. (10) indicates that this Kessler-based semi-analytical formulation is mathematically equivalent to that derived independently by Bassa et al.; specifically, see appendix A of reference [11].

The analysis software calculates the integrals over $\theta$ and $\phi$ in Eq. (10) numerically. However, care must be taken when calculating the inner integral over $\phi$, because it can potentially contain singularities at locations where ${w}_{j}=0$. (Note: these integrable singularities appear as bright bands in sky plots of constellations, such as those shown in Figs. 4, 11, 12 and 13 in Bassa et al. [11].) The implemented algorithm divides the $\phi$ integral into segments bounded by such singularities if and when necessary, and then evaluates each segmented integration using Matlab’s *integral* function, which accurately handles the singularities using an adaptive quadrature algorithm [26]. The algorithm also uses Matlab’s *integral* function to evaluate the outer $\theta$ integral.

**Fig. 2:**
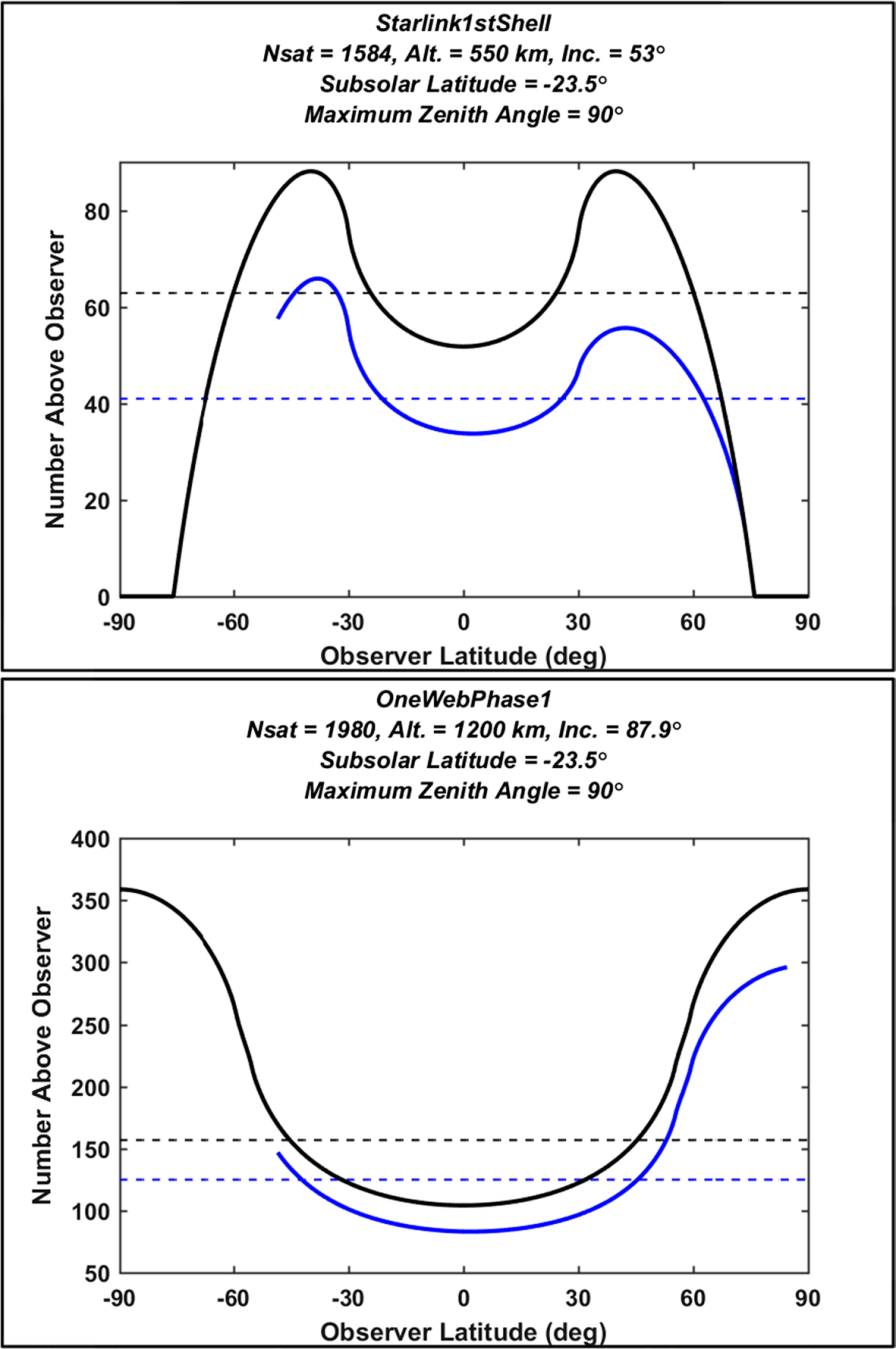
The number of visible and illuminated satellites as a function of observer latitude, for the first shell Starlink 1st Gen. constellation (top), and for the OneWeb Phase 1 constellation (bottom). The solid black lines plot the total number of visible satellites in the sky above ground-based observers. The solid blue lines plot the number also illuminated by the sun for a solar depression angle of 18° and a subsolar latitude of − 23.5°. The horizontal dashed lines show the corresponding uniform satellite distribution approximations

Figure 2 plots ${N}_{v}$ and ${N}_{i}$ values calculated using Eq. (10) for the first shell of the Starlink 1st Gen. constellation (top panel) and the OneWeb Phase 1 constellation (bottom panel). Specifically, the solid black lines show ${N}_{v}$ calculated as a function of observer latitude, plotted in a format used previously to illustrate global variations in these quantities [1, 2]. The solid blue lines show ${N}_{i}$ at the start and end of astronomical nighttime (i.e., when the SDA $\alpha$ = 18°) and for northern winter solstice observing conditions (i.e., for a subsolar latitude of ${\beta }_{s}$ = −23.5°). Note that for observer latitudes less than -48.5° and greater than 84.5° the SDA does not equal 18° for the entire 24-h period bracketing N winter solstice, as indicated by the solid blue curves in each plot. Figure 2 shows that both ${N}_{v}$ and ${N}_{i}$ vary significantly as a function of observer latitude for these two single-shell constellations, reaching peak values at northern and southern latitudes roughly equal to the shell’s inclination.

#### The Uniform Thin Shell Approximation

The dashed lines in Fig. 2 plot estimates for ${N}_{v}$ and ${N}_{i}$ calculated using a simpler approximation that distributes the constellation satellites in a uniform thin shell over all latitudes (see references [1] and [15] for more details on this approximation method). While this approach provides reasonable approximations for low latitude observers, it can significantly underestimate ${N}_{v}$ and ${N}_{i}$ at higher latitudes, as is apparent in the bottom panel of Fig. 2. For this reason, this analysis does not employ the uniform thin shell approximation for light pollution evaluation.

#### Comparisons of the Kessler-based Formulation and the Bassa et al. Formulation

As mentioned previously, the Kessler-based formulation presented in this study is mathematically equivalent to that derived independently by Bassa et al. [11]. Quantitative comparisons also indicate that the two software implementations accurately match one another. For example, for observations conducted from the Paranal observatory (located at latitude ${\beta }_{o}$ = − 25°) at equinox illumination conditions (i.e., for subsolar latitude ${\beta }_{s}$ = 0), the Kessler-based approach yields ${N}_{v}$ = 1398 for the combined Starlink 1st and 2nd Generation constellations, and ${N}_{v}$ = 651 for the combined OneWeb Phase 1 and 2 constellations. These values accurately match those plotted in Fig. 10 of Bassa et al. Similarly, for a solar depression angle of $\alpha$ = 18°, Paranal equinox observing conditions yield ${N}_{i}$ values of 781 and 524, respectively, for these two combined constellations, which again accurately match those plotted in Fig. 10 of Bassa et al.

### The Number of Brighter-Than-Recommended Satellites Above an Observer

The primary indicator used in this analysis to evaluate constellation light pollution is ${N}_{b}$, which represents the statistically expected number of satellites above a ground-based observer that are also brighter than the recommended threshold given by Eq. (1). The analysis formulates this indicator in order to measure the adverse impact of constellations on both naked eye observations of the sky, as well as on professional ground-based astronomy conducted in the visible and near-IR spectral bands. However, it is worth acknowledging that this single metric does not measure the entire set of adverse effects for all ground-based observational modalities, especially those conducted in different spectral bands, which require additional considerations. For instance, even in the visible and near-IR spectral bands, the slower angular velocity of satellites at higher altitudes leads to increased surface brightness on an image, because each satellite spends more time illuminating a given image pixel [1, 11]. Although the altitude dependence of the recommended brightness threshold in Eq. (1) partially accounts for this effect [3], angular rates are not otherwise included as part of the evaluation analysis. Despite these limitations, this analysis adopts ${N}_{b}$ as the primary light pollution indicator because it represents a consolidated metric that simultaneously accounts for the effects of constellation population, orbital distribution as well as brightness magnitude and variability.

Deriving a semi-analytical expression for ${N}_{b}$ first entails inserting another binary factor into the integrand of Eq. (5), which is equal to one if the satellite is brighter in reflected sunlight than the recommended brightness threshold and zero otherwise [15]. The derivation of ${N}_{b}$ then proceeds much as described previously for the quantities ${N}_{v}$ and ${N}_{i}$, and results in the following expression for the *j*th shell of the constellation

11 $${N}_{b,j}\approx \frac{{N}_{c,j}}{2{\pi }^{2}{a}_{j}}{\int }_{0}^{\pi /2}d\theta \left\{\frac{{s}_{\theta } \; {f}_{j}\left(\theta ,\kappa \right) {\varrho }^{2}\left({a}_{j},\theta \right)}{ {\left[{a}_{j}^{2}-{({R}_{e}{s}_{\theta })}^{2}\right]}^{1/2}}{\int }_{0}^{2\pi }d\phi \left[\frac{U\left({w}_{j}\right) {I}_{s}({{\varvec{r}}}_{j},{{\varvec{r}}}_{s})}{\sqrt{{w}_{j}}}\right]\right\}$$

with the total given by a sum over all constellation shells, ${N}_{b}={\sum }_{j}{N}_{b,j}$. Equation (11) is similar in form to Eq. (10), except that the integrand contains an additional function, ${f}_{j}\left(\theta ,\kappa \right)$, which represents the phase angle-averaged fraction of satellites in the *j*th shell observed at zenith angle $\theta$ that also have apparent magnitudes brighter than the recommended threshold, ${\mathbb{M}}({h}_{j})$, given by Eq. (1). As described below, this brighter-than-recommended fraction depends on both the observation zenith angle, $\theta$, and the atmospheric extinction coefficient, $\kappa$.

For a given constellation, ${N}_{b}$ depends on the time of the observation, the longitude and latitude of the observatory, as well as the extinction coefficient, i.e., ${N}_{b}={N}_{b}(t,{\lambda }_{o},{\beta }_{o},\kappa )$. Like ${N}_{i}$, during astronomical nighttime periods ${N}_{b}$ achieves its largest value for an SDA of $\alpha$ = 18°, and tends to decrease relatively quickly with increasing SDA for lower altitude constellations, but persist further into astronomical night for higher altitude constellations [15].

### Empirical Estimation of the Brighter-than-Recommended Fraction of Constellation Satellites

There are two primary ways to estimate the brighter-than-recommended fraction ${f}_{j}\left(\theta ,\kappa \right)$ for each shell of constellation satellites. The first is to build a software simulation model based on the constellation’s actual satellite design that uses realistic and accurate bi-directional reflectance distribution functions for the surface materials, as recommended in previous constellation studies [3, 7–10]. The second method entails empirically estimating ${f}_{j}\left(\theta ,\kappa \right)$ in a statistical manner, based on a representative distribution of photometric measurements [11, 15], which is the focus of this study.

This analysis estimates ${f}_{j}\left(\theta ,\kappa \right)$ using the data plotted in Fig. 1. Adjusting each photometric measurement to account for expected apparent magnitude changes due to observer-to-satellite range variations and atmospheric extinction [11] yields the following approximation

12 $${f}_{j}\left(\theta ,\kappa \right)=\frac{\sum_{k=1}^{{N}_{obs}}\left[U\left({\mathbb{M}}\left({h}_{j}\right)-{M}_{n,k}-5{\mathrm{log}}_{10}\left(\varrho \left({a}_{j},\theta \right)/{\rho }_{n}\right)-\kappa \mathcal{X}(\theta )\right)\right]}{{N}_{obs}}$$

In this equation, $U\left(x\right)$ again indicates the unit step function, ${M}_{n,k}$ the range-normalized magnitude derived from the *k*th photometric observation, and $\mathcal{X}(\theta )$ the airmass at zenith angle $\theta$. The numerator of this expression represents the number of observations with adjusted magnitudes that are brighter than the recommended threshold, ${\mathbb{M}}\left({h}_{j}\right)$, given by Eq. (1). Dividing by the total number of observations, ${N}_{obs}$, then provides an empirical estimate of the brighter-than-recommended fraction. This analysis uses a representative V-band extinction coefficient of $\kappa$ = 0.12 magnitudes/airmass [11], along with the airmass approximation of Rozenberg [27]

13 $$\mathcal{X}\left(\theta \right)\approx \frac{1}{\left[{c}_{\theta }+0.025 \mathrm{exp}(-11{c}_{\theta })\right]}$$

which yields $\mathcal{X}\left(0^\circ \right)\approx 1$, $\mathcal{X}\left(60^\circ \right)\approx 2$, and $\mathcal{X}\left(90^\circ \right)\approx 40$.

**Fig. 3:**
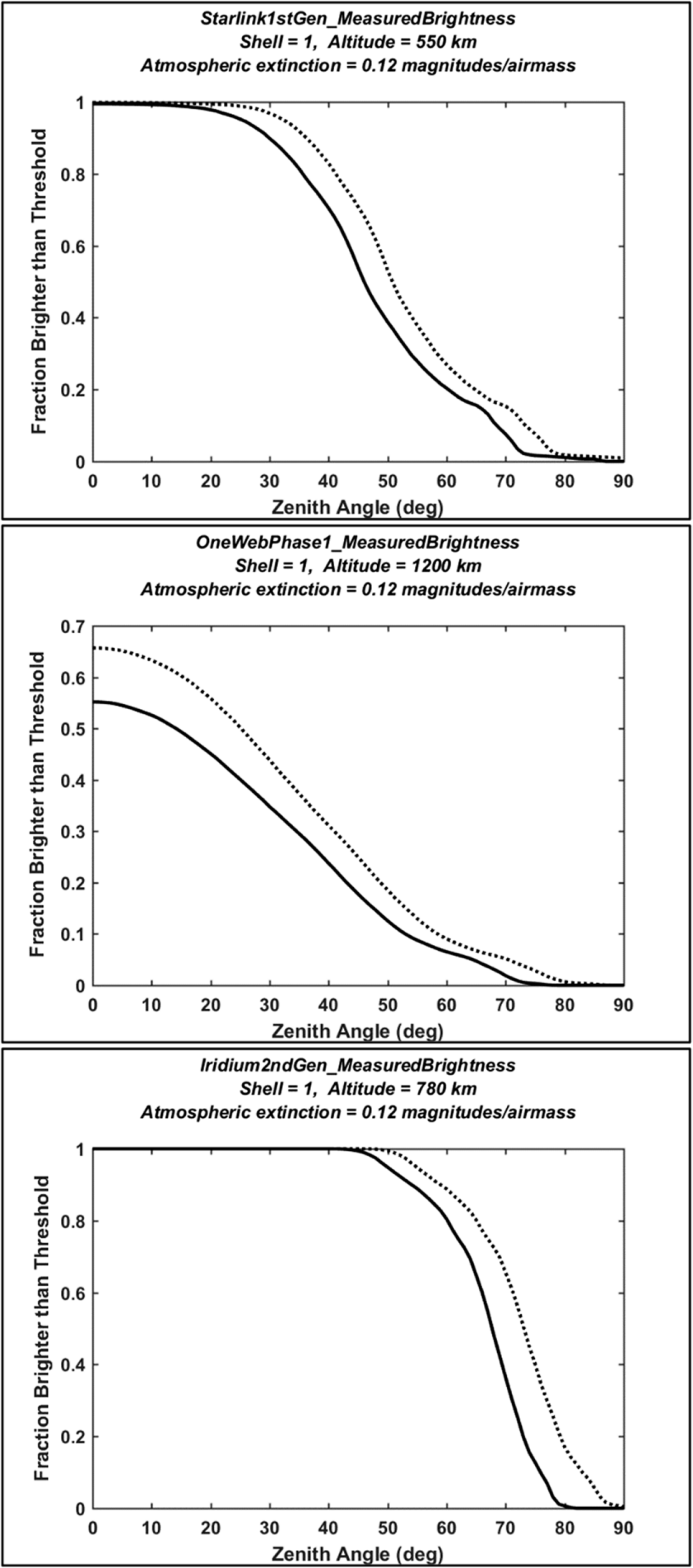
Empirically estimated fractions of the first shell of the Starlink 1st Gen., the OneWeb Phase 1 and the Iridium 2nd Gen. constellation satellites (top to bottom) that are brighter than the recommended threshold, plotted as a function of observation zenith angle. Dotted lines show estimates neglecting atmospheric extinction (i.e., for $\kappa$ = 0), and solid lines show estimates for an extinction coefficient of $\kappa$ = 0.12 magnitudes/airmass

Figure 3 plots ${f}_{j}\left(\theta ,\kappa \right)$ values estimated for the first shell of Starlink 1st Gen., the OneWeb Phase 1 and the Iridium 2nd Gen. constellations, calculated by applying Eq. (12) to the photometric data sets shown in Fig. 1. The decreasing trend of the curves indicates the effect of increasing observer-to-satellite range and airmass as a function of zenith angle. For $\theta =0$ and $\kappa$ = 0, the plotted ${f}_{j}\left(\theta ,\kappa \right)$ values equal the brighter-than-recommended fractions reported in Sect. 4, i.e., ${f}_{0}={f}_{j}\left(0, 0\right)$, which equal 99.9%, 65.7% and 100% for these three constellations, respectively. For $\theta =60^\circ$ and $\kappa$ = 0.12 magnitudes/airmass, these ${f}_{j}\left(\theta ,\kappa \right)$ values decrease to 20.4%, 6.5%, and 80.4%, respectively. For large zenith angles (i.e., $\theta \ge 75^\circ$), the brighter-than-recommended fractions decrease to relatively small values in all three cases, due to increasing range and especially atmospheric extinction.

### Yearly and Global Peak Values for the Number of Brighter-Than-Recommended Satellites

As mentioned previously, for a given constellation, the number of brighter-than-recommended satellites above a ground-based observer depends on four variables: ${N}_{b}={N}_{b}(t,{\lambda }_{o},{\beta }_{o},\kappa )$. This analysis uses a fixed extinction coefficient of $\kappa$ = 0.12 magnitudes/airmass, so this variable is suppressed hereafter for brevity. Also, instead of specifying the three variables, $(t,{\lambda }_{o},{\beta }_{o})$, the function ${N}_{b}$ can be calculated equivalently (i.e., with no loss of generality) as a function of three angles: the geocentric latitude of the observer, ${\beta }_{o},$ the subsolar geocentric latitude, ${\beta }_{s}$, and the solar depression angle, $\alpha$. This leads to the following alternative functional expression: ${N}_{b}={N}_{b}({\beta }_{o},{\beta }_{s},\alpha )$. During one full year, the subsolar latitude varies over the range − 23.5° ≤ ${\beta }_{s}$  ≤ 23.5°, and the maximum value over this range represents the yearly peak (YP) value

14 $${N}_{b}^{YP}({\beta }_{o},\alpha )=\underset{{|\beta }_{s}| \le 23.5^\circ }{\mathrm{max}}\left[{N}_{b}({\beta }_{o},{\beta }_{s},\alpha )\right]$$

This function is symmetric in observer latitude, i.e., ${N}_{b}^{YP}\left({\beta }_{o},\alpha \right)={N}_{b}^{YP}({-\beta }_{o},\alpha )$. The maximum of the yearly peak value over all latitudes represents the global peak (GP) value

15 $${N}_{b}^{GP}(\alpha )=\underset{|{\beta }_{o}|\le 90^\circ }{\mathrm{max}}\left[{N}_{b}^{YP}({\beta }_{o},\alpha )\right]$$

which is a function of the solar depression angle, $\alpha$. The analysis software determines the maxima in eqs. (14) and (15) numerically, using a one-dimensional bisection search algorithm in each case [28].

The global peak function given by Eq. (15) provides a highly consolidated way to measure visible and near-IR band light pollution for an entire constellation. For astronomical nighttime conditions, the largest value for this function occurs at an SDA of $\alpha$ = 18°, which is used to define an overall light pollution (LP) indicator, as follows

16 $${N}_{b}^{LP}={N}_{b}^{GP}(\alpha =18^\circ )$$

Notably, this nonnegative scalar depends on no remaining observational or environmental variables, so a single ${N}_{b}^{LP}$ value characterizes each constellation. This consolidated indicator facilitates quantitative comparisons of constellations with different numbers of shells deployed at different altitudes and inclinations. It also enables comparisons of existing and proposed constellations to one another, as well as trade-space studies of design-phase constellations. The ${N}_{b}^{LP}$ indicator is intrinsically conservative, because it represents the peak time-averaged number of brighter-than-recommended constellation satellites expected to occur above potential observer locations distributed around the globe and during astronomical nighttime periods that occur throughout an entire year. For these reasons, this analysis uses ${N}_{b}^{LP}$ as defined in Eq. (16) as the primary indicator to evaluate constellation light pollution.

In addition to the indicator given by Eq. (16), which represents the peak over all observer latitudes, the evaluation analysis uses three secondary indicators defined by dividing the globe into three latitude bands: low latitudes spanning $0\le \left|{\beta }_{o}\right|\le 23.5^\circ$, medium latitudes $23.5^\circ <\left|{\beta }_{o}\right|\le 66.5^\circ$, and high latitudes $66.5^\circ <\left|{\beta }_{o}\right|\le 90^\circ$.

#### Light Pollution Evaluation for the Starlink 1st Generation Constellation

**Fig. 4:**
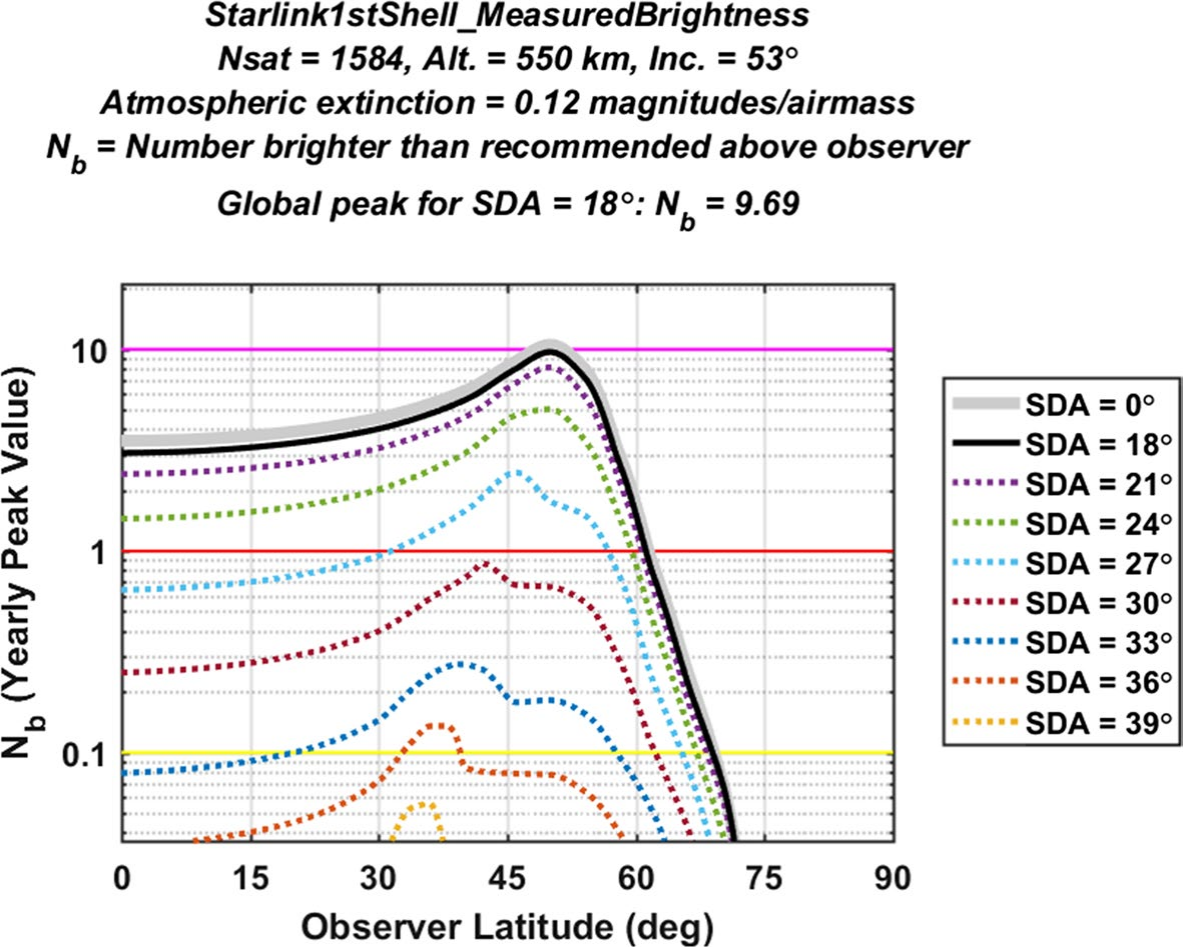
Yearly peak number of brighter-than-recommended satellites above ground-based observers estimated for the first shell of the Starlink 1st Gen. constellation. The black curve plots ${N}_{b}^{YP}$ as a function of observer latitude for SDA = 18°; the other curves plot ${N}_{b}^{YP}$ for other SDA values. The maximum point on the black curve represents the global peak, indicating an overall light pollution indicator value of ${N}_{b}^{LP}$ = 9.69

Figure 4 plots yearly peak light pollution levels as a function of observer latitude for the first shell of the Starlink 1st Gen. constellation. Specifically, the graph shows ${N}_{b}^{YP}$ curves calculated using Eq. (14), assuming only VisorSat design satellites as characterized by the empirical V-band range-normalized magnitude distribution shown in the top panel of Fig. 1. The solid black line shows the specific curve used to evaluate overall constellation light pollution, calculated using an SDA of $\alpha =18^\circ$ and an extinction coefficient of $\kappa =0.12$ magnitudes/airmass, as described earlier. The other curves plot ${N}_{b}^{YP}$ for different SDA angles, and indicate how much the peak light pollution levels persist into astronomical night. The maximum point on the black curve (located in this case at an observer latitude of ${\beta }_{o}\approx 50^\circ$) represents the global peak value, and corresponds to an overall light pollution indicator of ${N}_{b}^{LP}$ = 9.69 for this first shell of the Starlink constellation (which has been completely deployed at the time of this study).

The yellow, red and magenta horizontal lines plotted in Fig. 4 indicate different levels of constellation light pollution. Regions above the red line correspond to an expected yearly peak level of more than one brighter-than-recommended constellation satellites in the sky above ground-based observers. Similarly, regions above the magenta line correspond to a level of more than ten brighter-than-recommended satellites. Table 2 lists the four color-coded levels used in the analysis for light pollution evaluation, varying from very high down to low. For instance, the estimated value of ${N}_{b}^{LP}$ = 9.69 apparent in Fig. 4 indicates that the first shell of the Starlink 1st Gen. constellation by itself registers a “high” level of light pollution, corresponding to the red color code listed in Table 2.

**Table 2:**
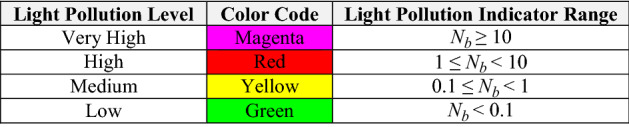
Color-coded constellation light pollution evaluation levels

**Fig. 5:**
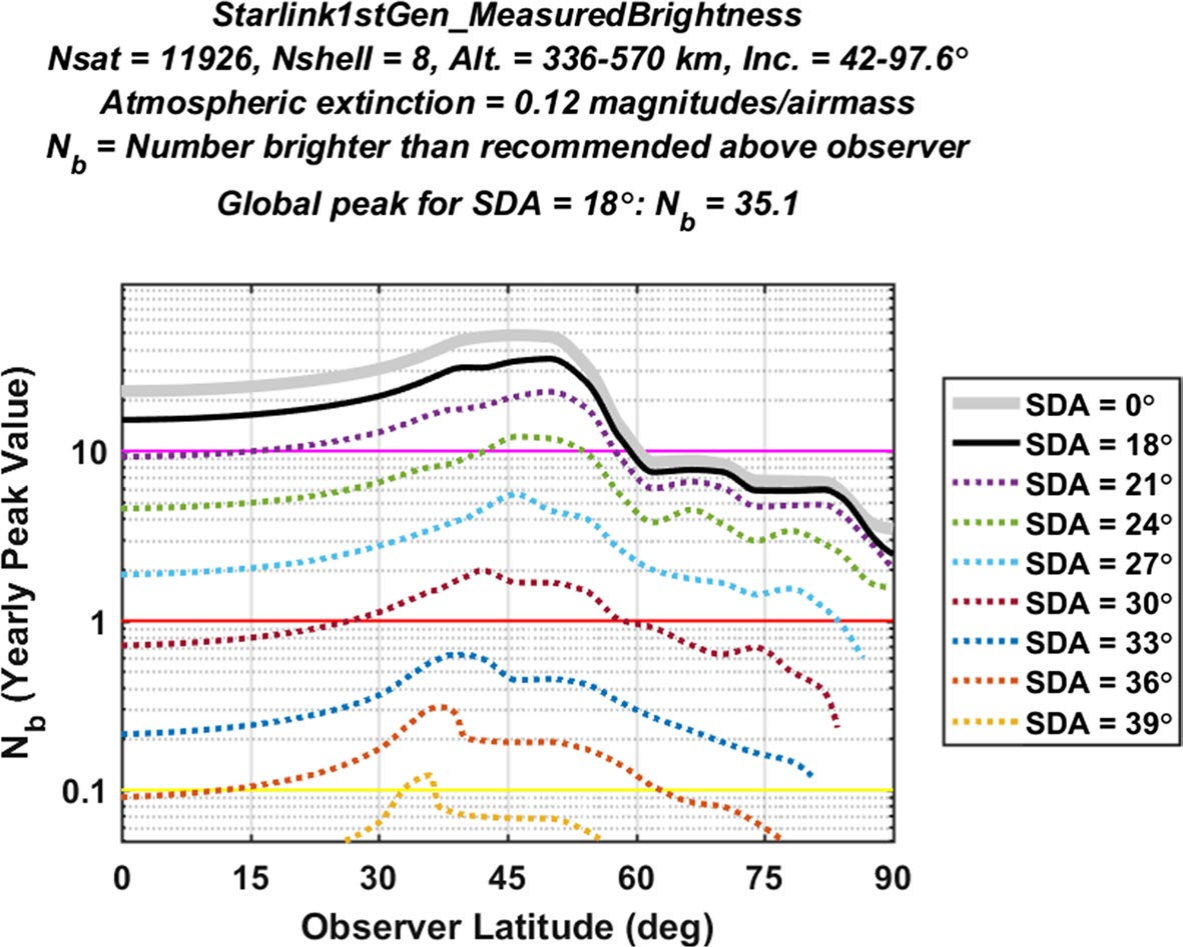
Yearly peak light pollution levels for the Starlink 1st Gen. constellation, plotted as a function of observer latitude. The peak of the black curve indicates an overall light pollution level of ${N}_{b}^{LP}$ = 35.1

Figure 5 shows the yearly peak light pollution level plot estimated for the Starlink 1st Generation constellation, again assuming only VisorSat design satellites. The analysis predicts ${N}_{b}^{LP}$ = 35.1, corresponding to a very high (i.e., magenta) overall light pollution level for this partially deployed large, multi-shell constellation. However, this predicted level could conceivably change, if the manufacturing design and/or the orbital distribution of the constellation satellites were to change significantly before complete deployment. This is not an unlikely possibility, as the SpaceX corporation has already changed satellite manufacturing designs in order to mitigate light pollution, which is how the VisorSat design model was introduced [7–10, 21, 22]. Section 6 below discusses methods to estimate light pollution indicators for such modified satellite designs, as well as for newly proposed constellations.

As mentioned previously, the consolidated light pollution indicator ${N}_{b}^{LP}$ does not uniformly measure the entire set of adverse effects for all ground-based sensor modalities. However, the benefits of reducing a constellation’s ${N}_{b}^{LP}$ level can be demonstrated quantitatively by estimating the associated improvements for wide-field astronomical imaging. As discussed by Bassa et al. [11], a 100 square-degree imager (e.g., with a 10° × 10° field of view) pointed in the zenith direction will experience a significant rate of contamination by satellite trails from a large bright constellation. Specifically, a single-shell constellation of 10,000 satellites deployed at 1000 km altitude and 53° inclination can potentially create 0.5 streaks per short-duration image for a sensor deployed a latitude of 30° (as shown in Fig. 3 of [11], and reproduced by the expressions presented in this analysis). For the Starlink 1st Gen. constellation, a similar analysis predicts that brighter-than-recommended satellites would produce a global-peak rate of 0.6 streaks per image during astronomical nighttime conditions for such a zenith-pointing wide-field imager. This means that, under certain conditions, streaks from brighter-than-recommended satellites would pollute more than half of all acquired images. However, if the light pollution indicator could be reduced from the magenta level of ${N}_{b}^{LP}$ = 35.1 down to a yellow or green level of ${N}_{b}^{LP}$ < 1, then brighter-than-recommended Starlink satellites would create < 0.02 streaks per image, meaning that fewer than one out of fifty images would be affected. Even though the exact quantitative improvements depend on both the astronomical observation modality and the constellation’s specific parameters, this example demonstrates the benefits of reducing the ${N}_{b}^{LP}$ indicator to below the magenta and red levels listed in Table 2.

**Fig. 6:**
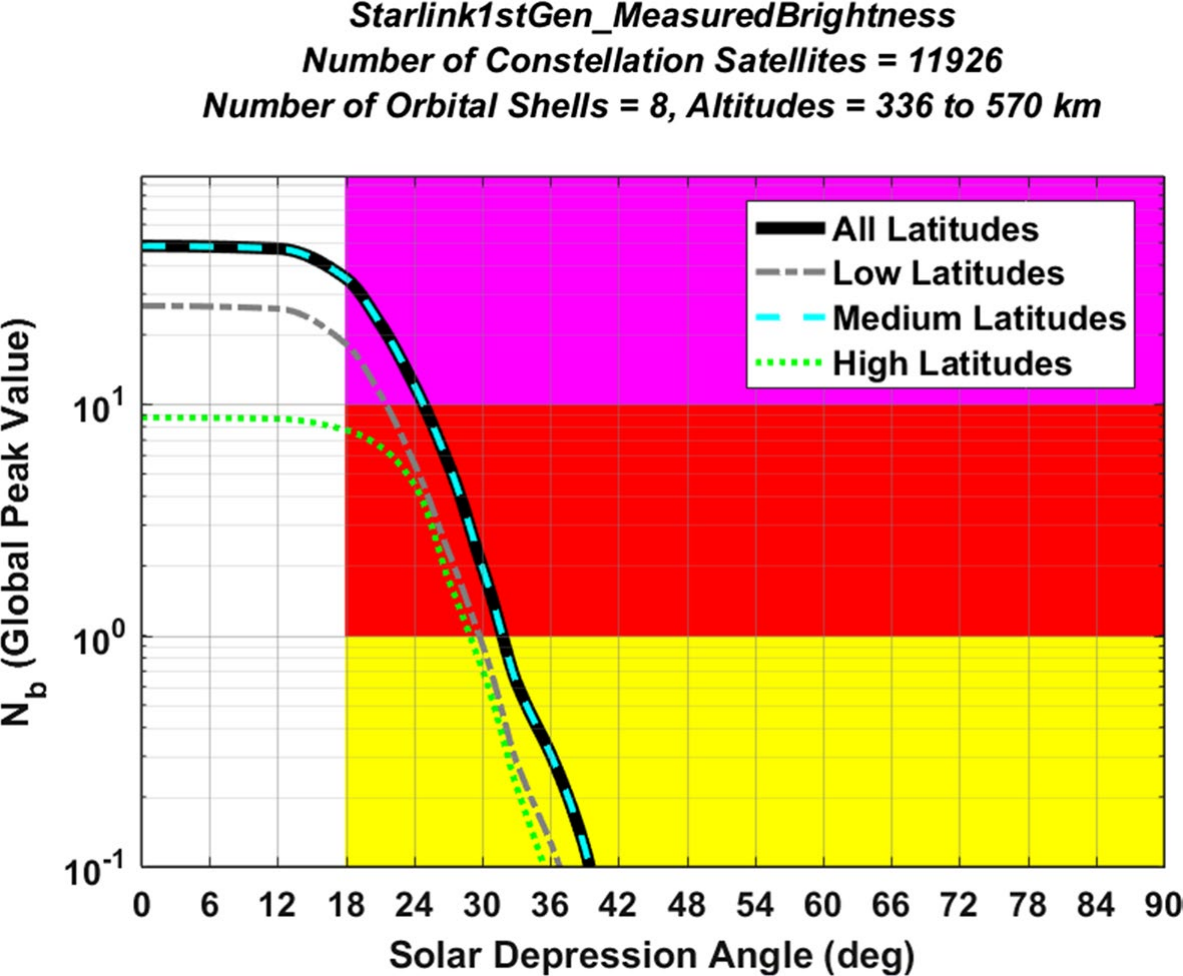
Global peak light pollution indicator levels for the Starlink 1st Gen. constellation, plotted as a function of solar depression angle. The solid black curve shows the global peak number of brighter-than-recommended satellites above ground-based observers distributed over the entire globe (i.e., ${N}_{b}^{GP}$ values). The other curves show light pollution levels for observers in low, medium and high latitude bands. Yellow, red, and magenta shading indicates regions evaluated to have medium, high and very high levels of light pollution, respectively

Figure 6 shows global peak light pollution levels predicted for the combined Starlink 1st Generation constellation plotted as a function of SDA. In particular, the solid black curve shows ${N}_{b}^{GP}$ values calculated using Eq. (15). The three other curves show light pollution levels for the low, medium and high latitude bands described previously. The color shading indicates regions evaluated to have elevated levels of light pollution, as listed in Table 2. For this constellation, global peak light pollution indicator levels occur within the medium latitude band, but are somewhat lower in the low latitude band, and lower yet in the high latitude band. Figure 6 also indicates that the Starlink 1st Gen. global light pollution indicator decreases to medium levels or below for SDA angles of $\alpha$ > 32°, and to low levels for $\alpha$ > 40°.

#### Light Pollution Evaluation for the OneWeb Constellation Phase 1 Constellation

**Fig. 7:**
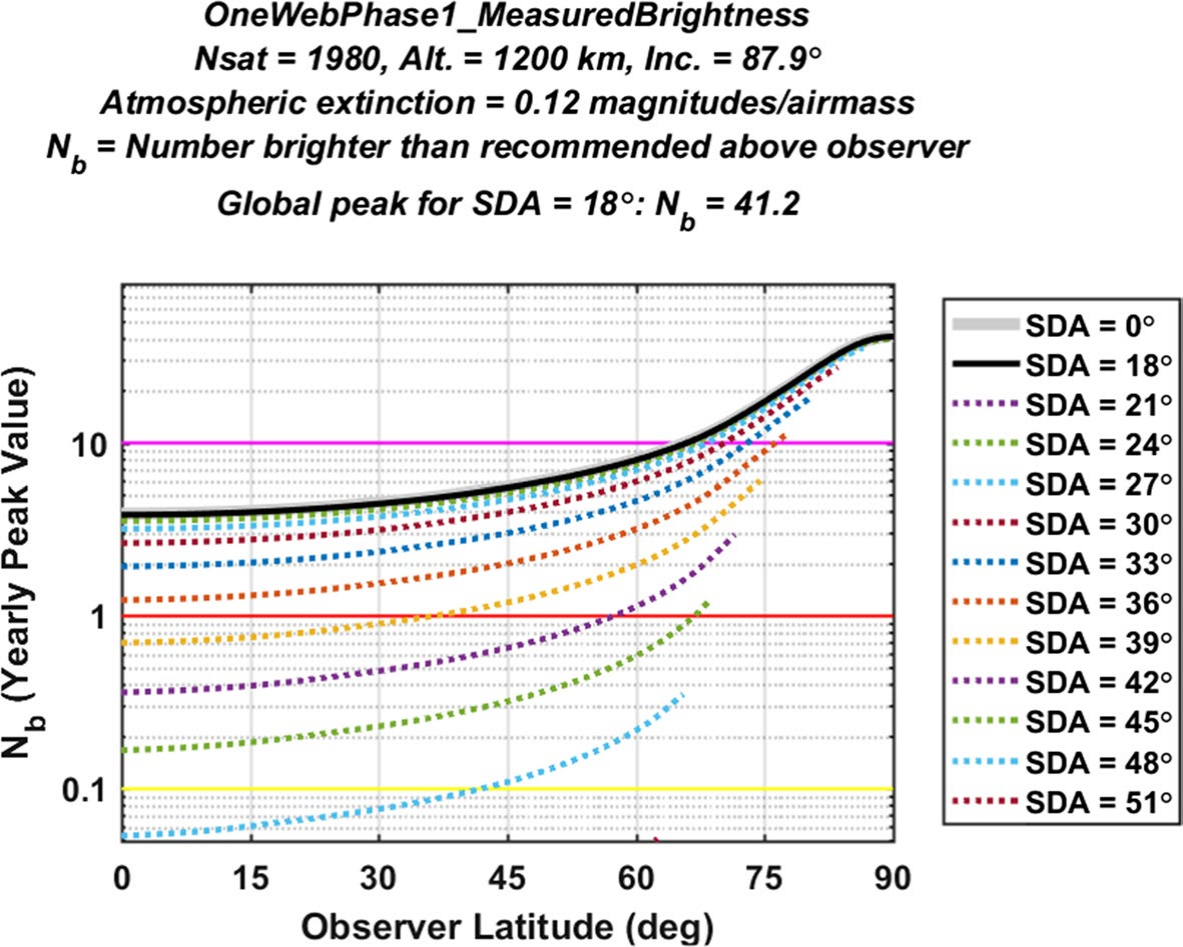
Yearly peak light pollution levels for the OneWeb Phase 1 constellation, plotted as a function of observer latitude. The peak of the black curve indicates an overall light pollution level of ${N}_{b}^{LP}$ = 41.2 (Color figure online)

**Fig. 8:**
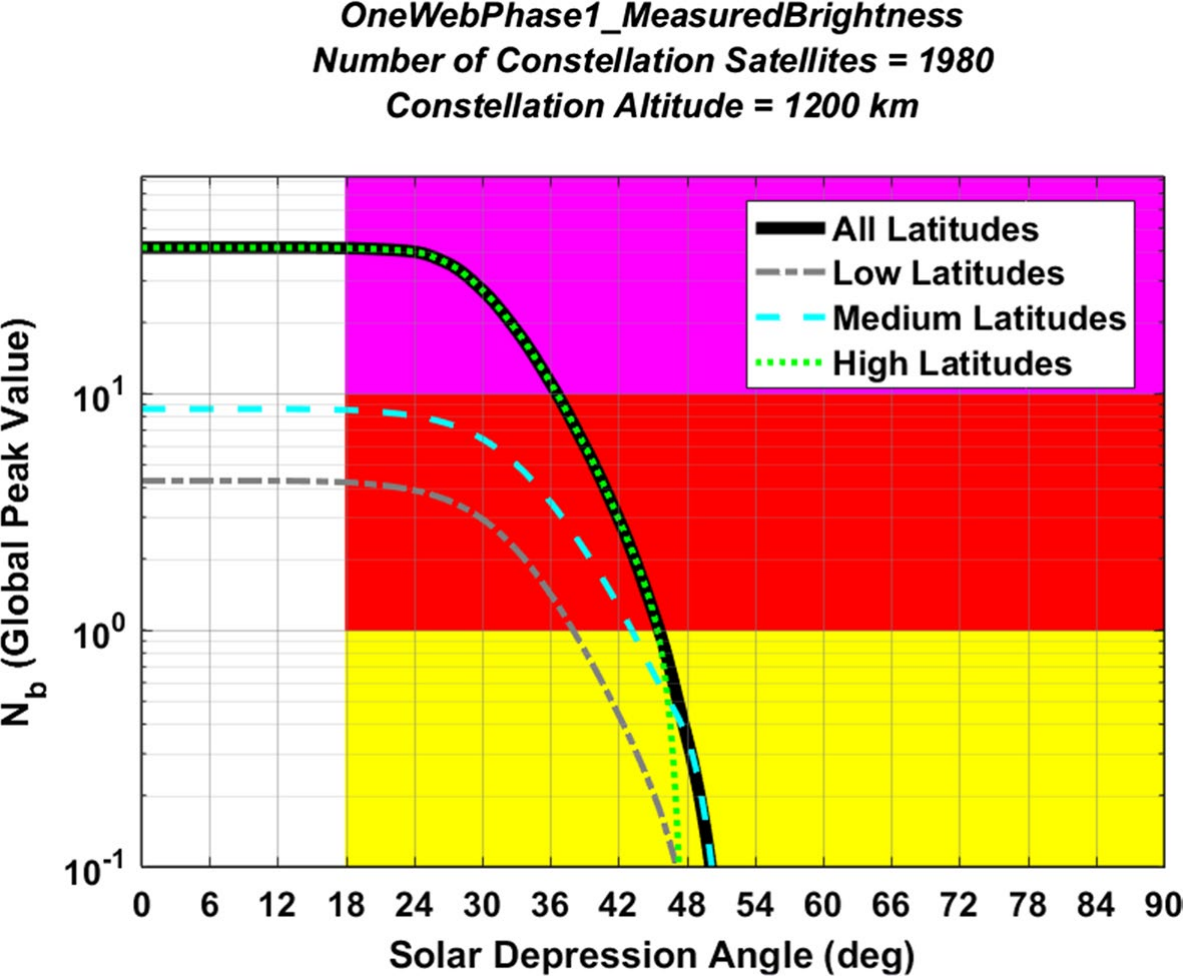
Global peak light pollution levels for the OneWeb Phase 1 constellation, plotted as a function of solar depression angle, indicating that the highest light pollution levels occurs at high latitudes

Figures 7 and 8 plot light pollution levels for the large OneWeb Phase 1 constellation in the same format used above for Figs. 5 and 6. Again, the analysis characterizes all of the satellites in this constellation using the empirical V-band magnitude distribution measured for currently orbiting OneWeb satellites, shown in the middle panel of Fig. 1. The peak of the solid black curve in Fig. 7 occurs at Earth’s poles, and indicates a very high evaluated light pollution level of ${N}_{b}^{LP}$ = 41.2 for this partially deployed constellation. Again, this evaluation could change if the design or the orbital distribution of the constellation satellites were to change significantly. Both Figs. 7 and 8 show the largest light pollution levels at high latitudes, which is due to the high inclination of this constellation. Figure 8 shows that the OneWeb Phase 1 global light pollution indicator decreases to medium levels or below for SDA angles of $\alpha$ > 46°, which means that high levels of light pollution for this constellation persists significantly further into astronomical night than for the lower-altitude Starlink constellation.

For the OneWeb Phase 1 constellation, the analysis indicates that brighter-than-recommended satellites would produce a global-peak rate of about 1.3 streaks per image for a 100 square-degree wide-field imager pointing in the zenith direction. However, if the OneWeb Phase 1 constellation light pollution indicator could be reduced from the magenta level of ${N}_{b}^{LP}$ = 41.2 down to a yellow or green level of ${N}_{b}^{LP}$ < 1, then brighter-than-recommended satellites would create < 0.03 streaks per image, again quantitatively demonstrating the benefits of reducing the ${N}_{b}^{LP}$ indicator for large constellations.

### Comparisons of Evaluated Light Pollution Levels for the Studied Constellations

**Fig. 9:**
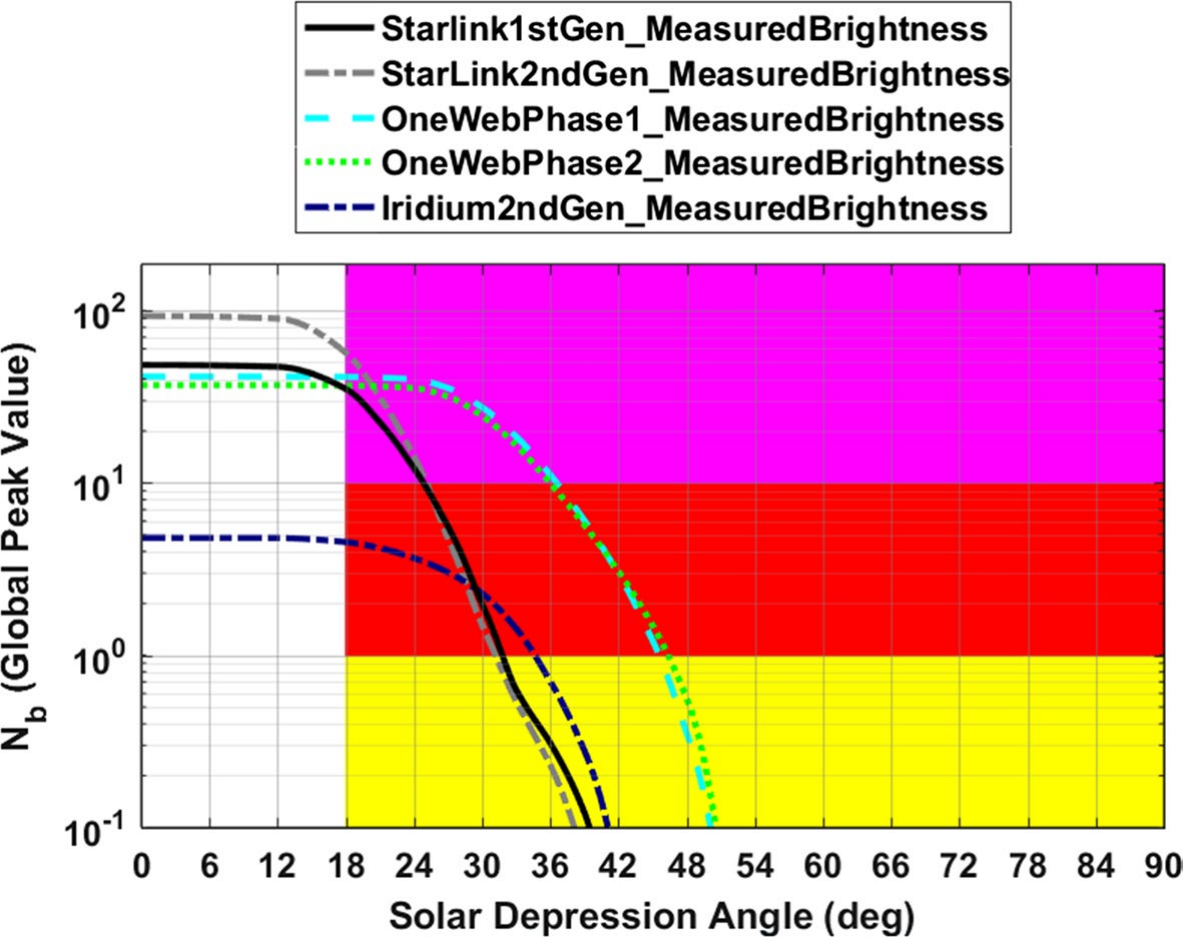
Semi-empirical light pollution levels for the constellations analyzed in this study. The evaluation indicates that the large Starlink and OneWeb constellations all have “very high” (i.e., magenta) light pollution levels, and the Iridium constellation has a “high” (i.e., red) level

Figure 9 compares global peak light pollution levels for the five constellations analyzed in this study. In all five cases, the evaluations use the brightness distributions measured for currently orbiting satellites shown in Fig. 1. The semi-empirical analysis indicates that the four populous Starlink and OneWeb constellations all have “very high” evaluated light pollution levels, because their curves pass through the magenta shaded region. As mentioned previously, elevated levels of light pollution for the two OneWeb constellations persist further into astronomical night than for the other, lower-altitude constellations.

Table 3 summarizes the color-coded semi-empirical light pollution indicators for all of the studied constellations, as well as the two hypothetical single bright satellites discussed in Sect. 3. As mentioned previously, the four populous Starlink and Iridium constellations all register very high (magenta) light pollution levels, with ${N}_{b}^{LP}$ ≥ 10. Among these, the most populous Starlink 2nd Gen. constellation has the highest light pollution indicator of all, ${N}_{b}^{LP}$ = 56.9, which is more than a factor of ten larger than that of less populous Iridium 2nd Gen. constellation, ${N}_{b}^{LP}$ = 4.53. The representative low inclination single satellite registers a low (i.e., green) light pollution level in all latitude bands, with ${N}_{b}^{LP}$ = 0.048; this means that it would require a constellation of about 1/${N}_{b}^{LP}$ ≈ 21 such bright satellites to register a high (red) evaluated level, and a constellation of about 210 to register a very high (magenta) level. The representative high inclination single satellite registers a medium (yellow) light pollution level, ${N}_{b}^{LP}$ = 0.117, within the high latitude band; it would require a constellation of ≈9 such satellites to register a red level, and ≈90 to register a magenta level.

**Table 3:**
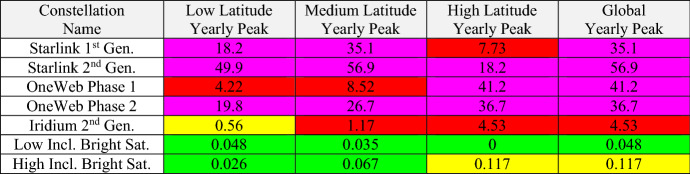
Light pollution evaluation levels for the studied constellations

The preponderance of red- and magenta-level entries among the Starlink and OneWeb constellations in Table 3 indicates that these large constellations (which are still mostly undeployed at the time this article is being written) represent very high (magenta-level) light pollution threats. Notably, the evaluation even assesses the significantly less populous Iridium 2nd Gen. constellation to produce high (red-level) light pollution levels, except for at low latitudes. This assessment reflects the relatively high satellite brightnesses measured for this fully deployed constellation, as shown in the bottom panel of Fig. 1. If another constellation similar to Iridium 2nd Gen. were to be proposed, then the evaluation analysis could be used indicate how much the brightnesses of the new satellite would need to be reduced in order to improve the light pollution indicator to a more acceptable level, as described in more detail below.

## Evaluating Light Pollution Risks for New or Proposed Constellations

Plotting the global-peak light pollution indicator in the format used in Fig. 9 provides a graphical means to evaluate the potential impact of new or proposed constellations, both in comparison to existing constellations, and in an absolute sense as well. Specifically, the process entails first predicting ${N}_{b}^{GP}(\alpha )$ for the new constellation, and then applying a two-part test to determine the associated effects on ground-based observations.

### Comparing a New or Proposed Constellation to an Ensemble of Existing Constellations

This section demonstrates the evaluation process using a hypothetical scenario, by seeking an answer to the following query. If the Starlink 1st Gen. and OneWeb Phase 1 constellations were the only two that currently existed, would adding a one of the other three as a hypothetical new constellation significantly increase the risk to visible and near-IR ground-based astronomy? Of course, any new set of satellites launched into orbit will have some effect, but this analysis tests for two specific types of light pollution impact, as evaluated by answering two quantitatively addressable questions:

1. Would the new constellation have the largest ${N}_{b}^{GP}$ indicator at any time during astronomical night?
2. Would the new constellation produce a red or magenta overall light pollution level, with ${N}_{b}^{LP}$ ≥ 1?

The first question probes how the new constellation compares to others, in a relative sense. The second addresses how the new constellation performs with respect to the absolute, predefined standards given in Table 2. An answer of yes to either of these questions indicates that the new constellation fails the light pollution evaluation.

A careful inspection of Fig. 9 indicates that the proposed Starlink 2nd Gen. constellation would fail both light pollution impact tests in this hypothetical scenario. It fails the first because, during the small portion of astronomical night when the SDA is in the range 18° ≤ $\alpha$  ≤ 20°, the Starlink 2nd Gen. constellation would be predicted to have a larger ${N}_{b}^{GP}$ value than either of the pre-existing Starlink 1st Gen. or OneWeb Phase 1 constellations. It also fails the second test because at $\alpha$ = 18° the predicted ${N}_{b}^{GP}$ curve for the Starlink 2nd Gen. constellation lies within the magenta shaded region, implying an overall light pollution indicator of ${N}_{b}^{LP}$ = 56.9, which is much larger than the red-level cutoff value of one.

Similarly, Fig. 9 indicates that the proposed OneWeb Phase 1 constellation would also fail both tests in this hypothetical scenario. It fails the first because, during the portion of astronomical night with $\alpha$ ≥ 42°, it has a larger predicted ${N}_{b}^{GP}$ value than any pre-existing constellation. It also fails the second test because ${N}_{b}^{LP}$ = 36.7, which again is much larger than the red-level cutoff.

Finally, Fig. 9 indicates that if the Iridium 2nd Gen. constellation were to be proposed as a new constellation in this hypothetical scenario it would pass the first test, but would fail the second test with ${N}_{b}^{LP}$ = 4.53.

These three hypothetical comparisons demonstrate how the two-part test provides the means to evaluate quantitatively the potential for a new constellation to be become a significant new source of light pollution for ground-based observers. For these examples, all three of the proposed constellations would produce levels of light pollution evaluated to be unacceptable. The recommendation for each would be to change the proposed constellation’s population, orbital distribution, and/or satellite design, in order to pass both parts of the test, and thereby mitigate the risk to ground-based visible and near-IR astronomy.

### Estimating Brightnesses for Future Proposed Constellation Satellites

The two-part test described in the previous section relies on measuring or predicting the statistical distribution of constellation satellite brightnesses. More specifically, the evaluation process requires estimates for the function ${f}_{j}\left(\theta ,\kappa \right)$, which statistically characterizes the fraction of brighter-than-recommended satellites in the *j*th shell of the constellation, as described in Sect. 5.3. There are two basic ways to estimate these brighter-than-recommended fractions. The first is to build a software simulation model based on the constellation’s actual satellite design that uses realistic and accurate bi-directional reflectance distribution functions for the surface materials, as recommended and described in previous constellation studies [3, 7–10]. This provides the means to simulate a statistically representative ensemble of ground-based light-curves, and estimate from those the associated ${f}_{j}\left(\theta ,\kappa \right)$ values. However, this labor-intensive method may not be feasible in all cases, especially during a constellation’s early planning stages. The second method entails estimating empirically ${f}_{j}\left(\theta ,\kappa \right)$ in a statistical manner, based on actual photometric measurements [11, 15]. For existing constellations, measurements of the constellation satellites themselves (such as those plotted in Fig. 1) provide the required data, as described previously. This section describes two extensions to this semi-empirical method. The first assesses the benefits and feasibility of relatively minor modifications to current constellation satellite designs by applying a fixed offset to the observed distribution of visual magnitudes. The second focuses on entirely new proposed constellations, and entails using observations of a set of analog objects (such as orbiting test prototypes, or other similarly designed satellites), adjusted to account for known differences between the analog and constellation satellites [15].

#### Required Brightness Adjustments for Currently Orbiting Constellation Satellites

As an example of the first method, consider the ongoing efforts by the SpaceX company to reduce the brightness of the Starlink satellites [8] by incorporating relatively minor modifications to current manufacturing designs. This effort originally led the introduction of the VisorSat design, which deployed a sun-blocking visor [22] demonstrated to improve median V-band range-normalized magnitudes by about $\Delta M$ = 1.3 relative to the previous design [19, 21]. One rough way to approximate the effect of such future design changes for currently orbiting constellation satellites (such as Starlink or OneWeb) is to apply a fixed magnitude offset, $\Delta M$, to the measured distribution of range-normalized brightnesses, which reflects the desired improvement from the planned design changes

17 $${M}_{n,k}^{new}={M}_{n,k}+\Delta M$$

with ${M}_{n,k}^{new}$ indicating the range-normalized magnitude for the redesigned satellite, which is fainter than the *k*th measurement for the current design, ${M}_{n,k}$. Inserting these new range-normalized magnitudes into Eq. (12) allows the estimation of new ${f}_{j}\left(\theta ,\kappa \right)$ fractions, and associated light pollution indicator levels. This approximation provides a rough but simple means of assessing the benefits and feasibility of satellite design improvements.

For instance, the overall light pollution indicator of ${N}_{b}^{LP}$ = 36.7 for the proposed OneWeb Phase 2 constellation listed in Table 3 assumes the deployment of 6372 OneWeb satellites as they are currently designed. Reanalyzing the constellation using Eq. (17) indicates that $\Delta M$ ≈ 1.7 would be required to reduce the assessment down to a more acceptable value of ${N}_{b}^{LP}$ ≈ 1, a challenging but likely feasible level of brightness reduction. This means that redesigning the OneWeb satellites alone could feasibly achieve a significant light pollution improvement, even without reducing the numbers of satellites in the proposed future Phase 2 constellation.

However, the situation is not the same for the proposed Starlink 2nd Gen. constellation, which is predicted to have a ${N}_{b}^{LP}$ = 56.9 based on the deployment of 30,000 satellites. Equation (17) indicates that an improvement of $\Delta M$ ≈ 2.7 would be required to achieve ${N}_{b}^{LP}$ ≈ 1, a much more challenging and potentially infeasible level of brightness reduction over the VisorSat design. In this case, however, the Starlink 2nd Gen. constellation could further reduce the predicted ${N}_{b}^{LP}$ indicator by also decreasing the large proposed population of 30,000 satellites.

#### Adjusting the Brightness Distributions of Currently Orbiting Analog Satellites

Combining a detailed satellite brightness model and photometric simulation software provides the means to estimate brightness distributions for new constellations [3, 7–10]. This approach may not be feasible, however, for constellations in their early phases of planning and design. In these cases, however, photometric observations of currently orbiting analog satellites offer an empirical method to approximate roughly constellation light pollution levels. The estimation process requires varying degrees of adjustments to account for the differences between the analog and constellation satellites [15]. For instance, if the proposed satellite system plans to use identical or nearly identical satellite designs and flight control profiles, then no adjustments to the range-normalized magnitude distribution would be required. Notably, this study has already used this method, by applying the brightness distributions observed for currently orbiting Starlink and OneWeb satellites to estimate the light pollution levels for future stages of those constellations. Similarly, if a proposed constellation intends to use the same exact design used by a set of orbiting satellites, but with the linear dimensions systematically changed by a scale factor ${S}_{f}$, then Eq. (17) can be used to account for the differences, using the fixed magnitude offset $\Delta M=-2.5{\mathrm{log}}_{10}({S}_{f}^{2})$, with the factor of ${S}_{f}^{2}$ accounting for the change in satellite projected areas. This approach would be applicable to a proposed constellation that has launched a set of prototype satellites for testing, identical to the intended production design, but reduced in linear size by the scale factor ${S}_{f}$. This method provides empirical estimates of the proposed constellation’s light pollution levels, based on ground-based measurements of the brightness distribution of the orbiting prototypes.

For example, consider the hypothetical example of a proposed Iridium 3rd Gen. constellation, comprising the same satellite population and orbital distribution as the currently deployed Iridium 2nd Gen. constellation. As mentioned previously, current Iridium 2nd Gen. satellites exceed the recommended brightness threshold to such an extent that even this 75 satellite constellation has a red-level light pollution indicator of ${N}_{b}^{LP}$ = 4.53. However, if the proposed Iridium 3rd Gen. satellites were reduced in linear dimension by a scale factor of ${S}_{f}$ ≈ 0.5, but otherwise used identical designs and flight control profiles, then the brightness distribution would shift by $\Delta M$ ≈ 1.5, which in turn would improve the light pollution indicator to below the red level. Considering the current Iridium design’s relatively large bus size of the of 3.1 m × 2.4 m × 1.5 m [29], such a size reduction would likely be feasible to achieve especially as micro- and nano-satellite capabilities continue to improve.

**Fig. 10:**
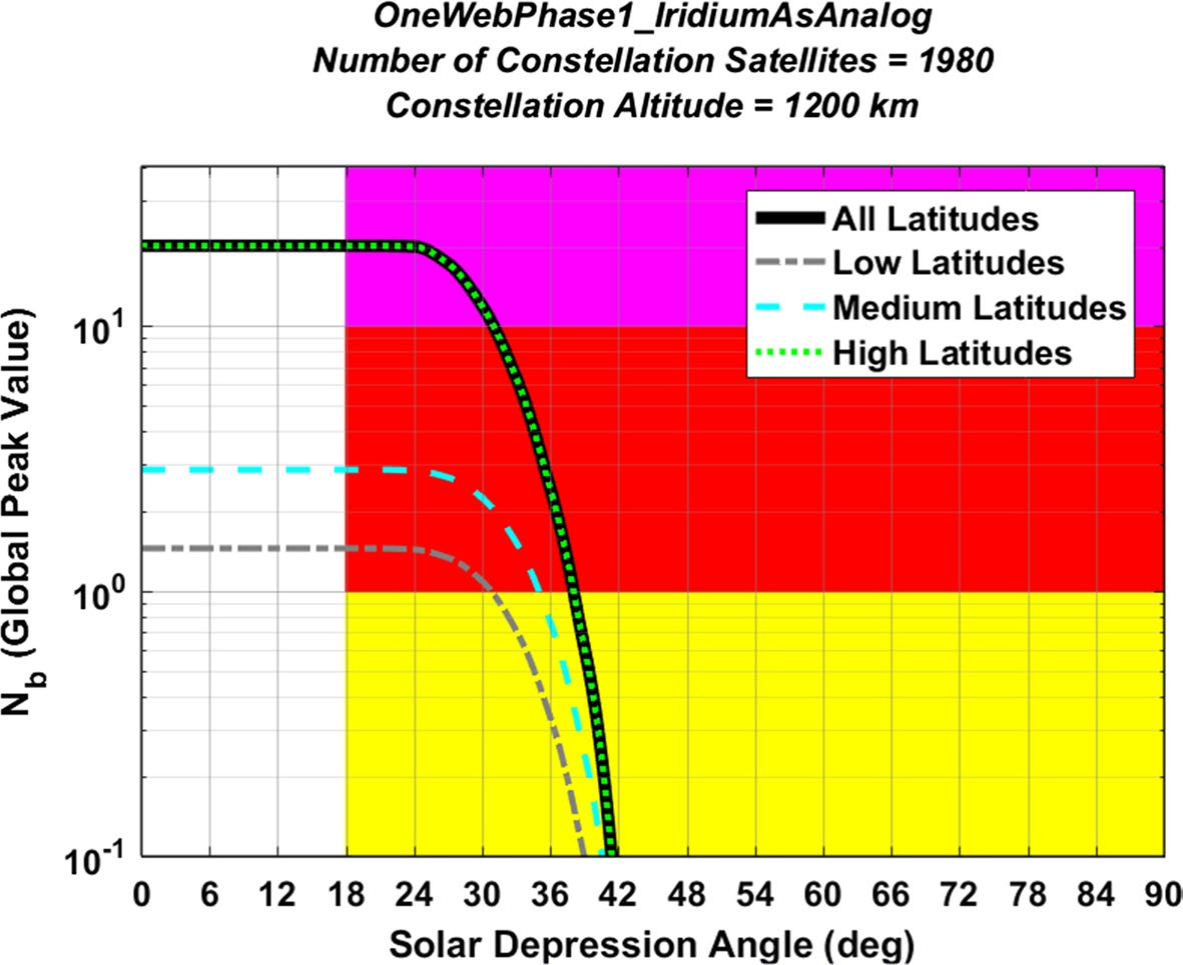
Global peak light pollution levels for the OneWeb Phase 1 constellation estimated by using Iridium 2nd Gen. satellites as analog objects

The existence of more significant design differences between the proposed and analog satellites introduces more uncertainty into the process, but still provides a rough means to estimate light pollution levels. To demonstrate the feasibility and limitations of the approach, this study uses the Iridium 2nd Generation satellites as analog objects in order to approximate light pollution levels for the OneWeb Phase 1 constellation. The manufacturing designs for these two satellites are roughly similar: both comprise a nadir-facing, roughly box-shaped bus with articulating solar panels mounted on either side [29, 30]. However, the finer details of these two independently manufactured satellite designs differ more significantly, such as the composition of the surface materials, as well as the shape and orientation of ground-tracking antennas. This analysis neglects these finer details and assumes that the photometric fluxes of the two satellite models scale roughly in proportion to their nadir-facing areas. With this simplification, the magnitude offset in Eq. (17) becomes $\Delta M\approx -2.5{\mathrm{log}}_{10}({A}_{nadir}/{A}_{nadir}^{analog})$ For Iridium 2nd Gen. analog satellites ${A}_{nadir}^{analog}\approx$ 7.4 m^2^, which corresponds to the 2.4 m by 3.1 m nadir-facing side of the bus [29]. For OneWeb satellites ${A}_{nadir}\approx$ 1.9 m^2^, corresponding to the 1.0 m by 1.3 m nadir-facing bus side, plus two ground-pointing parabolic antennas each estimated to have a radius of 0.3 m [30]. Applying this $\Delta M$ offset to the Iridium 2nd Gen. photometric data plotted in Fig. 1 allows the Iridium satellites to be used as analog objects for the OneWeb constellation. The analysis results in the global-peak indicator estimates plotted in Fig. 10. Comparing Fig. 10 to Fig. 8 indicates that the Iridium-as-analog method predicts light pollution indicator levels that are roughly a factor of two lower than the more accurate values based on actual OneWeb satellite data. Specifically, the analog satellite method yields an overall light pollution level of ${N}_{b}^{LP}\approx$ 20.1, compared to the more accurate value of ${N}_{b}^{LP}$ = 41.2. This level of inaccuracy likely reflects the fact that the approximation only accounts for the nadir-facing areas of the objects, neglecting contributions from the other surfaces, as well as differences in surface material reflectivities (i.e., albedos). However, despite these limitations, the broad conclusions of the two evaluations are consistent. Both predict very high (magenta) overall light pollution levels for the OneWeb Phase 1 constellation at high latitudes, along with high (red) levels at medium and low latitudes. Both also predict that high levels of light pollution persist significantly further into astronomical night for the OneWeb constellation than for the lower-altitude Starlink constellation.

Preliminary analysis indicates that accounting for material reflectivity (i.e., albedo) differences on the nadir-facing sides of the Iridium and OneWeb satellites improves the accuracy of the analog approximation somewhat. However, studies of other analog satellite cases indicates that the light pollution indicator prediction accuracy of the method is limited to roughly a factor of two, unless the two satellite designs are very similar.

## Discussion and Conclusions

This study formulates three semi-analytical indicators designed to evaluate the impact of satellite constellations on ground-based astronomy. The first of these, the time-averaged number of visible satellites above ground-based observers, ${N}_{v}$, represents the statistically expected number above the local horizon, which can be used to assess the impact of a constellation’s brightness in spectral bands not dominated by reflected sunlight (e.g., radio and thermal-IR) and due to occultation events. The second indicator, the expected number of satellites that are also illuminated by the sun, ${N}_{i}$, indicates a constellation’s potential impact on ground-based observations conducted in the visible and near-IR spectral bands. However, this indicator counts very bright and relatively dim satellites with equal weight, even though those that cause the most concern to the astronomical community have V-band magnitudes brighter than the threshold given in Eq. (1), as recommended in the *SatCon-1 Workshop Report* [3]. To address this most concerning population, this study formulates a third light pollution indicator, ${N}_{b}$, the time-averaged number of brighter-than-recommended constellation satellites above ground-based observers. The formulation derives each of the ${N}_{v}$, ${N}_{i}$, and ${N}_{b}$ indicators as statistical expectation values, based on spatial distributions for constellation satellites estimated using the analytical Kessler probability density function [25], which results in semi-analytical expressions for each, calculated using two-dimensional numerical integration. Constellations with multiple orbital altitude shells require an additional summation. Analysis demonstrates that the Kessler-based, numerical integration approach formulated for ${N}_{v}$ and ${N}_{i}$ in this study is equivalent to the independently derived semi-analytical method of Bassa et al. [11].

The primary light pollution indicator used in this analysis, ${N}_{b}$, simultaneously incorporates the effects of constellation population and orbital distribution, as well as satellite brightness and associated variability. This study focuses on empirical methods of estimating this light pollution indicator. For existing constellations, actual photometric brightness measurements provide the necessary data. For proposed constellations, adjusting photometric observations of a set of analog satellites provides rough but worthwhile approximations. The evaluations presented in this analysis use a limited set of MMT system photometric observations [18], but could also use observations from other sources, such as the SatHub data repository [8]. Ideally, such data sources would provide information on non-detections and associated upper-limits, to enable use of the Kaplan–Meier method [23] to improve the semi-empirical estimates.

For a given constellation and atmospheric extinction coefficient, the number of brighter-than-recommended satellites depends on the latitude of the observer, the sub-solar latitude, the solar depression angle, i.e., ${N}_{b}={N}_{b}({\beta }_{o},{\beta }_{s},\alpha )$. The maximum value of this three-dimensional function represents the primary, overall constellation light pollution indicator formulated in this analysis, ${N}_{b}^{LP}$, an intrinsically conservative metric that corresponds to the peak number of brighter-than-recommended constellation satellites expected to occur above all potential observer locations distributed around the globe, and during all astronomical nighttime periods throughout a year,. This single scalar quantity characterizes an entire constellation, analytically accounting for the number, altitude, and inclination of satellites deployed within each orbital shell, and empirically accounting for each shell’s brightness distribution.

A two-part constellation evaluation process based on the number of brighter-than-recommended satellites indicator provides a quantitative method of comparing the impact that different constellations have on ground-based visible and near-IR band astronomical observations. Specifically, graphical comparison of global-peak ${N}_{b}^{GP}(\alpha )$ curves (as in Fig. 9) provides a means to evaluate constellation light pollutions levels based on both relative and absolute criteria. Table 2 list the specific color-coded absolute criteria adopted for this study, which could be adjusted to make the evaluation process more or less conservative. In this analysis, constellations with an overall light pollution indicator exceeding one (i.e., ${N}_{b}^{LP}\ge 1$) are ranked as having high light pollution levels; those with ${N}_{b}^{LP}\ge 10$ are ranked as very high.

The evaluation process predicts that all four of the large planned constellations studied in this analysis, Starlink 1st and 2nd Gen. and OneWeb Phase 1 and 2, will produce very high light pollution levels when fully deployed, based on the photometric observations of currently orbiting satellites. More specifically, for each of these large constellations, 35 or more satellites bright enough to interfere with ground-based astronomy will be expected in the sky above some point on the Earth during astronomical nighttime periods. Notably, these very high light pollution predictions could be improved by reducing the brightness and/or number of constellation satellites. In some cases (e.g., for the 6372 satellite OneWeb Phase 2 constellation), realistically achievable reductions in the brightness of current-design satellites (by using optimal sun shading, darker surface materials, etc.) could feasibly reduce the light pollution indicator to a more acceptable level. In other cases, (e.g., the 30,000 satellite Starlink 2nd Gen. constellation), more drastic design changes might be required (e.g., a significant reduction overall satellite size), along with a significant decrease in the population of constellation satellites.

## Data Availability

MMT photometric temporal light-curve data used for the constellations studied in this analysis (as plotted in Figure 1), were obtained from the http://mmt9.ru/satellites/ website, which also makes available similar data for other satellites. The CARA light pollution evaluation software entitled “EvaluateConstellation” is currently being reviewed and approved to post at the NASA CARA Software Repository at the https://github.com/nasa/CARA_Analysis_Tools website. For all other data and software requests please contact the author.

## References

[1] Hainaut OP, Williams AP (2020). Impact of satellite constellations on astronomical observations with ESO telescopes in the visible and infrared domain. A&A.

[2] McDowell JC (2020). The Low Earth Orbit satellite population and impacts of the SpaceX Starlink constellation. Astrophys. J. Lett..

[3] Walker, C., et al.: Impact of Satellite Constellations on Optical Astronomy and Recommendations Toward Mitigations, SatCon-1 Workshop Report, NSF NOIRLab, Aug 2020

[4] Seitzer, P.: Large Constellations of LEO Satellites and Astronomy. In: The 2020 Technical AMOS Conference, Sep 2020

[5] Massey R, Lucatello S, Benveuti P (2020). The challenge of satellite megaconstellations. Nat. Astron..

[6] Venkatesan A, Lowenthal J, Parvath P, Vidaurri M (2020). The impact of satellite constellations on space as ancestral global commons. Nat. Astron..

[7] Walker, C., et al.: Report and Recommendations, Dark and Quiet Skies for Science and Society, United Nations Office for Outer Space Affairs, On-line Workshop Report, Jun, 2021

[8] Walker, C., et al.: Executive Summary, Report of the SATCON2 Workshop, NSF NOIRLab, AURA and AAS, Jul 12–16, 2021

[9] Hall, J., Walker, C. Rawls, M. L., McDowell, J., Seaman, R., Venkatesan, A., Green R., Krafton K., Parriot, J.: SatCon2 Working Group Reports, On-line report, 2021. https://noirlab.edu/public/media/archives/techdocs/pdf/techdoc033.pdf

[10] Walker C (2021). Working Group Reports.

[11] Bassa CG, Hainaut OR, Galadi-Enriquez D (2022). Analytical simulations of the effect of satellite constellations on optical and near-infrared observations. A&A.

[12] Boley AC, Byers M (2021). Satellite mega-constellations create risks in Low Earth Orbit, the atmosphere and on Earth. Sci. Rep..

[13] Newman LK (2010). The NASA robotic conjunction assessment process: overview and operational experiences. Acta Astronaut..

[14] National Aeronautics and Space Administration, NASA Spacecraft Conjunction Assessment and Collision Avoidance Best Practices Handbook, NASA/SP-20205011318, Dec 2020

[15] Hall, D.: Semi-Empirical Metrics to Measure the Effects of large Satellite Constellations on Astronomy. In: The 2021 AMOS Technical Conference Proceedings, Kihei, HI. (2021)

[16] Graham, W.: Iridium Next-5 Satellites Ride to Orbit on SpaceX Falcon 9, NASA SpaceFlight.com, Mar 28, 2018 (retrieved from archive copy from the original web posting on Mar 1, 2021)

[17] Vallado DA (2001). Fundamentals of Astrodynamics and Applications.

[18] Karpov S, Beskin G, Biryukov A (2018). Photometric calibration of a wide-field sky survey data from Mini-MegaTORTORA. Astron. Nachr..

[19] Malama, A.: Starlink satellite brightness – characterized from 100,000 visible light magnitudes. arXiv e-prints arXiv:2101.09735 (2021)

[20] Malama, A.: The brightness of OneWeb satellites. arXiv e-prints arXiv:2012.05100 (2020)

[21] Malama, A.: The brightness of VisorSat-design Starlink satellites. arXiv e-prints arXiv:2101.00375 (2021)

[22] Cole RE (2020). A sky brightness model for the starlink “VisorSat” spacecraft. Res. Notes AAS.

[23] Fiegelson ED, Nelson PI (1985). Statistical methods for astronomical data with upper limits. I. Univariate distributions. Astrophys. J..

[24] Hall, D., Calef, B., Knox, K., Bolden, M., Kervin, P.: Separating Attitude and Shape Effects for Non-Resolvable Objects. In: The 2007 AMOS Technical Conference Proceedings, Kihei, HI, 2007

[25] Kessler DJ (1981). Derivation of the collision probability between orbiting objects: the lifetimes of Jupiter’s outer moons. Icarus.

[26] Shapine LF (2008). Vectorized adaptive quadrature in MATLAB. J. Comput. Appl. Math..

[27] Rozenberg, G.V.: Twilight: A Study in Atmospheric Optics, Plenum Press, Translated from the Russian by R.B. Rodman. (1966)

[28] Press WH, Teukolsky SA, Vettering WT, Flannery BB (1992). Numerical Recipes in FORTRAN: The Art of Scientific Computing.

[29] SpaceFlight 101.: Iridium-Next Satellite, from the https://spaceflight101.com/spacecraft/iridium-next webpage retrieved Jul 14, 2022

[30] eoPortal Directory.: OneWeb Minisatellite Constellation for Global Internet Service, from the https://directory.eoportal.org/web/eoportal/satellite-missions/o/oneweb webpage retrieved Jul 14, 2022

